# Fibroblast adipogenesis restricts hemangioma growth through a PPARγ/Hippo-YAP/TAZ axis that promotes endothelial apoptosis and cell cycle arrest

**DOI:** 10.1038/s41420-026-03176-x

**Published:** 2026-06-17

**Authors:** Sheng-Ming Qu, Bing Wang

**Affiliations:** Department of Dermatology, Second Hospital of Jilin University, Changchun, China

**Keywords:** Cell biology, Cell signalling

## Abstract

Infantile hemangioma (HEM) is the most common benign vascular tumor in infancy and is characterized by a unique progression pattern involving rapid proliferation followed by spontaneous involution. Increasing evidence suggests that the tumor microenvironment plays a critical role in regulating endothelial cell behavior during HEM development. In this study, we investigated how fibroblast (FB) phenotypic remodeling influences hemangioma endothelial cell (HemEC) function. Through single-cell RNA sequencing analysis, we identified extensive communication between FBs and ECs within HEM tissues. Functional experiments revealed that adipogenically differentiated FBs markedly suppressed HemEC proliferation, migration, invasion, and tube formation while promoting apoptosis. Transcriptomic analysis further demonstrated that this inhibitory effect was mediated by activation of the Hippo-YAP/TAZ signaling pathway in ECs. Mechanistically, adipogenic differentiation significantly upregulated PPARG expression in FBs, which in turn triggered Hippo pathway activation in ECs, leading to YAP/TAZ phosphorylation and nuclear exclusion. Importantly, PPARG silencing in FBs or pharmacological inhibition of the Hippo pathway reversed these effects. Collectively, our findings reveal a previously unrecognized microenvironmental regulatory mechanism in which PPARγ-driven fibroblast adipogenesis suppresses HEM progression through activation of the Hippo-YAP/TAZ pathway in endothelial cells. This study provides new insights into stromal-endothelial communication in HEM and suggests that the PPARγ–Hippo signaling axis may represent a potential therapeutic target.

**Figure:**
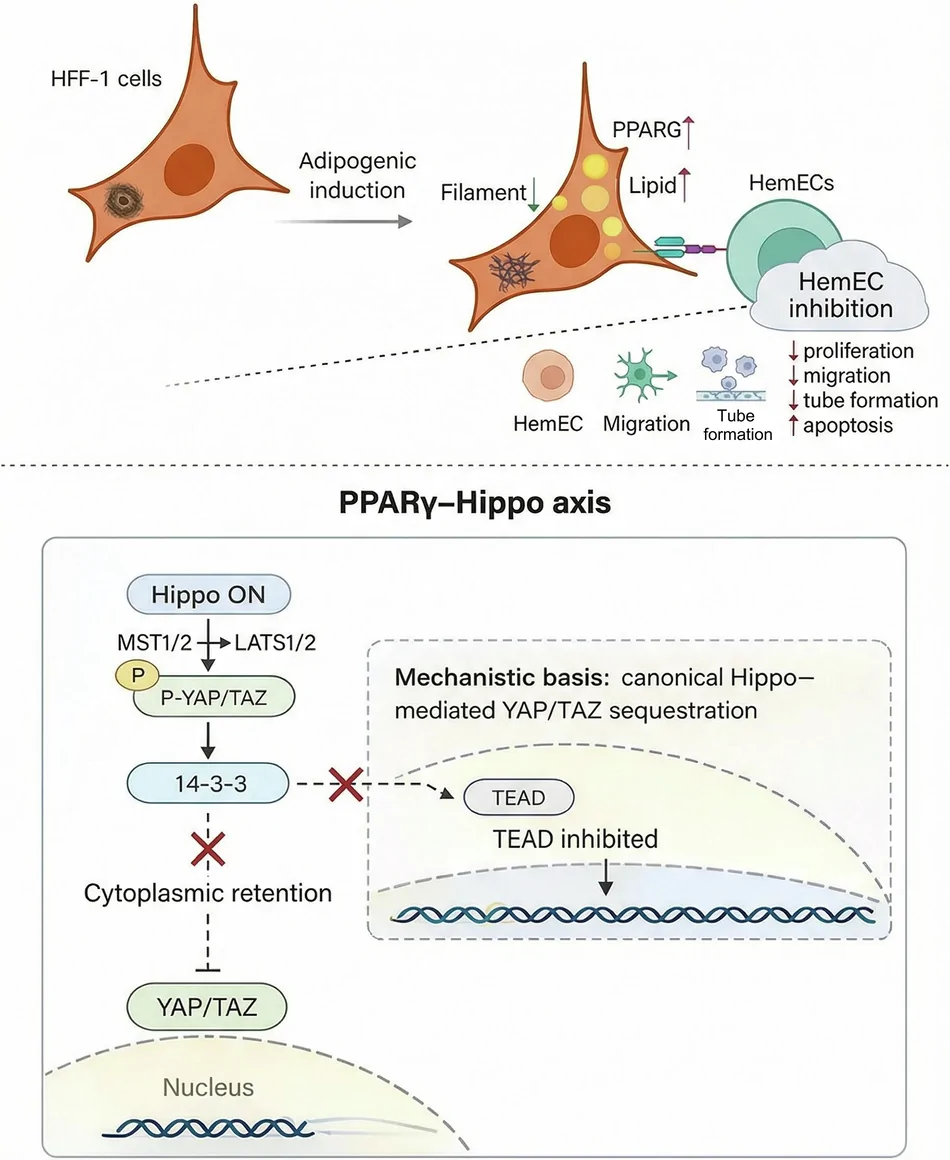
Proposed molecular mechanism by which PPARγ-mediated FB adipogenic differentiation regulates Hippo-YAP/TAZ signaling activity in HemECs. Note: Created in Biorender.

Proposed molecular mechanism by which PPARγ-mediated FB adipogenic differentiation regulates Hippo-YAP/TAZ signaling activity in HemECs. Note: Created in Biorender.

## Introduction

Hemangioma (HEM) is one of the most prevalent benign vascular tumors in infants, with an incidence rate ranging from 4% to 10% in newborns, warranting significant clinical attention [1, 2]. Characteristically, HEM emerges shortly after birth, followed by a rapid proliferation phase and a slow involution phase, with some cases completely regressing over several years [3, 4]. However, a substantial number of patients experience persistent progression of HEM, which can lead to local tissue invasion, organ compression, and complications such as ulceration and bleeding, significantly impacting the physiological function and quality of life of affected children [5, 6]. Understanding the molecular mechanisms underlying the development and progression of HEM to identify new therapeutic targets has become an urgent scientific challenge.

The formation and progression of HEM are not solely the result of abnormal proliferation of endothelial cells (ECs) but are closely associated with the complex interactions among various cell types within the tumor microenvironment [7, 8]. Extensive research has demonstrated that fibroblasts (FBs) play a pivotal role in angiogenesis and tumor progression, as their secreted cytokines and extracellular matrix (ECM) components can significantly influence EC behavior [9, 10]. Recent evidence indicates that the abundance and activity of fibroblasts within HEM tissues are closely associated with the proliferative state of the disease, suggesting that these cells play a critical role in the progression of HEM [11]. However, current studies on the function of FBs in HEM remain relatively limited, and the specific mechanisms by which they regulate EC fate under different conditions have yet to be fully elucidated. In recent years, with the advancement of single-cell RNA sequencing (scRNA-seq) technology, significant cellular heterogeneity within HEM tissues has been recognized, providing new research tools for further revealing intercellular communication networks [12]. Exploring the interaction patterns and molecular basis between FBs and ECs is crucial for understanding the pathogenesis of HEM and offers potential breakthroughs for optimizing therapeutic strategies.

The metabolic state of cells within the tumor microenvironment profoundly influences their function [13, 14]. Previous studies have demonstrated that metabolic reprogramming is not only a hallmark of malignant tumor cells but also plays a significant role in benign tumors such as HEM [15]. Under specific conditions, FBs can undergo adipogenic differentiation, a process that may alter local lipid metabolism levels and indirectly regulate the proliferation, migration, and survival of HemECs [16, 17]. PPARγ, as a central transcription factor in lipid metabolism and adipogenic differentiation, can drive FBs from an active proliferative state to an adipogenic state and plays a crucial role in angiogenesis and tissue repair across various tissues [18, 19]. Therefore, we hypothesize that, within the specific pathological context of HEM, PPARγ-mediated adipogenic differentiation of FBs represents a critical mechanism for regulating EC function.

The Hippo-YAP/TAZ signaling pathway has emerged as an important regulator of cell proliferation, apoptosis, and tissue homeostasis in recent years [20]. As transcriptional co-activators, YAP and TAZ regulate the expression of genes involved in cell growth and angiogenesis [21, 22]. Dysregulation of YAP/TAZ activity has been implicated in vascular abnormalities and tumor-associated angiogenesis [23, 24]. Despite these findings, the potential involvement of Hippo signaling in the regulation of hemangioma endothelial cell behavior remains poorly understood. In particular, whether microenvironmental factors such as fibroblast phenotypic remodeling influence Hippo signaling in endothelial cells has not yet been clarified.

Therefore, the present study aimed to elucidate how fibroblast phenotypic remodeling influences endothelial cell behavior during HEM progression. By integrating single-cell transcriptomic analysis with in vitro functional experiments, we investigated the regulatory role of fibroblast adipogenic differentiation in modulating endothelial cell function. In particular, we focused on PPARγ-mediated adipogenic differentiation in fibroblasts and examined its potential relationship with Hippo-YAP/TAZ signaling in endothelial cells. Through this approach, we sought to establish a mechanistic framework explaining how stromal cell metabolic reprogramming regulates endothelial cell fate in HEM.

## Results

### scRNA-seq reveals prominent fibroblast–endothelial communication in HEM tissues

**Fig. 1:**
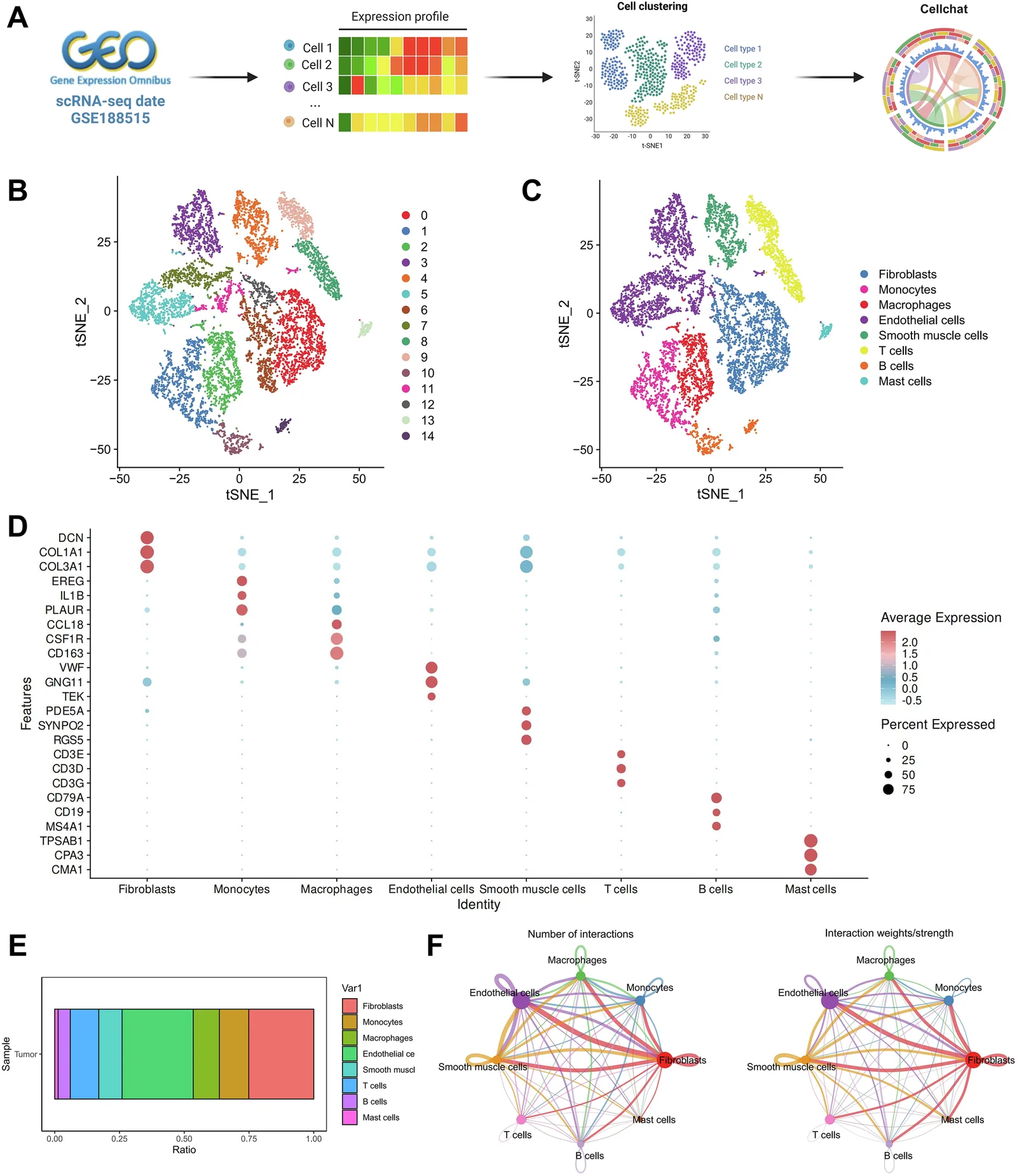
scRNA-seq reveals cellular heterogeneity within the HEM tumor microenvironment. **A** Workflow of scRNA-seq data analysis from the GEO dataset GSE188515 (*n* = 1), created with Biorender. **B** t-SNE visualization of clustering results, showing the spatial aggregation and distribution of cells, with each color representing a distinct cluster. **C** Cell type annotation based on t-SNE clustering, where each color denotes a specific cellular subpopulation. **D** Expression patterns of selected lineage-specific marker genes across different subpopulations, with deeper red indicating higher average expression and larger circles reflecting a greater proportion of cells expressing the gene. **E** Proportional distribution of the identified cellular subpopulations, with each color corresponding to a different cell type. **F** Circos plot illustrating the number and strength of intercellular communications among eight subpopulations, where line thickness indicates the quantity or intensity of signaling interactions.

To characterize the cellular composition of the HEM microenvironment and identify potential stromal–endothelial interactions, we first performed single-cell RNA sequencing (scRNA-seq) analysis of HEM tumor tissues. To investigate the cellular heterogeneity within HEM tumor tissues, scRNA-seq data derived from the central tumor region of patients with HEM were obtained from the GEO database for comprehensive analysis (Fig. 1A). The objective was to identify key factors influencing HEM onset and progression. Initially, low-quality cells were eliminated based on nFeature_RNA, nCount_RNA, and percent.mt from the scRNA-seq dataset (Fig. S1A). The filtered data revealed a correlation coefficient of *r* = −0.24 between nCount_RNA and percent.mt, and r = 0.94 between nCount_RNA and nFeature_RNA (Fig. S1B). Subsequently, the CellCycleScoring function was employed to compute cell cycle phases (Fig. S1C), and PCs were ranked by standard deviation, with PC_1 to PC_20 sufficiently capturing the information of highly variable genes (Fig. S1D). Nonlinear dimensionality reduction was performed using the t-SNE algorithm based on the top 20 PCs, followed by clustering using the clustree package at various resolutions (Fig. S1E). The resolution of 0.4 was selected, resulting in the division of all cells into 15 clusters (Fig. 1B). Next, cell type annotation was performed by referencing the CellMarker database [25], data from the Human Cell Atlas [26], and previously reported cell type-specific markers in the literature. As a result, eight distinct cell types were identified: FB, monocyte, macrophage, EC, smooth muscle cell, T cell, B cell, and mast cell (Fig. 1C).

The accuracy of the clustering annotation was validated by examining the expression of cell lineage-specific marker genes. For FB, marker genes included *COL3A1*, *COL1A1*, and *DCN* [27, 28]. Monocyte markers were *PLAUR*, *IL1B*, and *EREG* [29, 30]. Macrophage markers included *CD163*, *CSF1R*, and *CCL18* [29, 30]. EC markers were *TEK*, *GNG11*, and *VWF* [28, 31]. Smooth muscle cell markers included *RGS5*, *SYNPO2*, and *PDE5A* [32]. T cell markers were *CD3G*, *CD3D*, and *CD3E* [32]. B cell markers included *MS4A1*, *CD19*, and *CD79A* [28, 32]. Mast cell markers were *CMA1*, *CPA3*, and *TPSAB1* [32] (Fig. 1D). Additionally, the t-SNE expression profiles of these eight cell marker genes were presented (Fig. S2A).

The annotated cell proportions within the samples indicated a predominance of ECs and FBs (Fig. 1E). To further elucidate the signaling mechanisms between different cell populations and their potential roles in therapeutic response, the “CellChat” analytical framework was applied. This framework systematically assessed changes in intercellular communication networks based on ligand-receptor (L-R) interactions. Results indicated that the strongest interactions occurred between FB and EC subpopulations (Fig. 1F, Fig. S2B).

This comprehensive scRNA-seq analysis of HEM tumor tissues elucidated the cellular composition of the tumor microenvironment, highlighting the significant presence of ECs and FBs, and the pronounced communication between these two cell subpopulations.

### Fibroblast adipogenic differentiation suppresses HemEC proliferation, migration, and angiogenic capacity

HEMs are common benign tumors characterized by significant adipose tissue accumulation during their involution phase, suggesting that adipogenic differentiation plays a crucial role in their natural regression [33]. Studies have indicated that FBs possess adipogenic potential [34]; however, the regulatory mechanisms of FBs on HemECs activity remain unclear. Thus, this study investigates the role of FB adipogenic differentiation in regulating HemEC activity.

To investigate whether fibroblast adipogenic differentiation influences endothelial cell behavior, we established an adipogenic differentiation model in HFF-1 fibroblasts and examined its effects on HemEC function using a co-culture system. Using HFF-1 cells as a model, we established an adipogenic differentiation induction system and identified morphological changes during FB adipogenic differentiation (Fig. S3A). HFF-1 cells were divided into Control and Adipogenesis groups, with the latter induced for 7 days using adipogenic differentiation agents (Insulin, Dexamethasone, IBMX, and Indomethacin). Microscopic observation revealed that cells in the Adipogenesis group exhibited increased size, clear cell edges, and substantial cytoplasmic lipid droplet accumulation (Fig. S3B). Oil Red O (ORO) Staining confirmed the formation of lipid droplets, showing a significant increase in ORO-positive areas and lipid accumulation in Adipogenesis group cells (Fig. S3C), with a marked upregulation in OD quantitative analysis (Fig. S3D). Furthermore, LipidTOX immunofluorescence (IF) staining demonstrated a notable increase in the number and fluorescence intensity of lipid droplets in the Adipogenesis group compared to the Control group (Fig. S3E). IF analysis of actin filaments (ACTA2) in FBs indicated that, compared to the Control group, the Adipogenesis group exhibited sparser and more disordered filament structures with significantly reduced fluorescence intensity (Fig. S3F). In addition, Western blot analysis demonstrated that the expression levels of the adipogenic marker proteins PARIS, PGC-1α, and PPARγ were significantly upregulated in HFF-1 cells in the Adipogenesis group (Fig. S3G). These results indicate that FBs were successfully induced to undergo adipogenic differentiation.

**Fig. 2:**
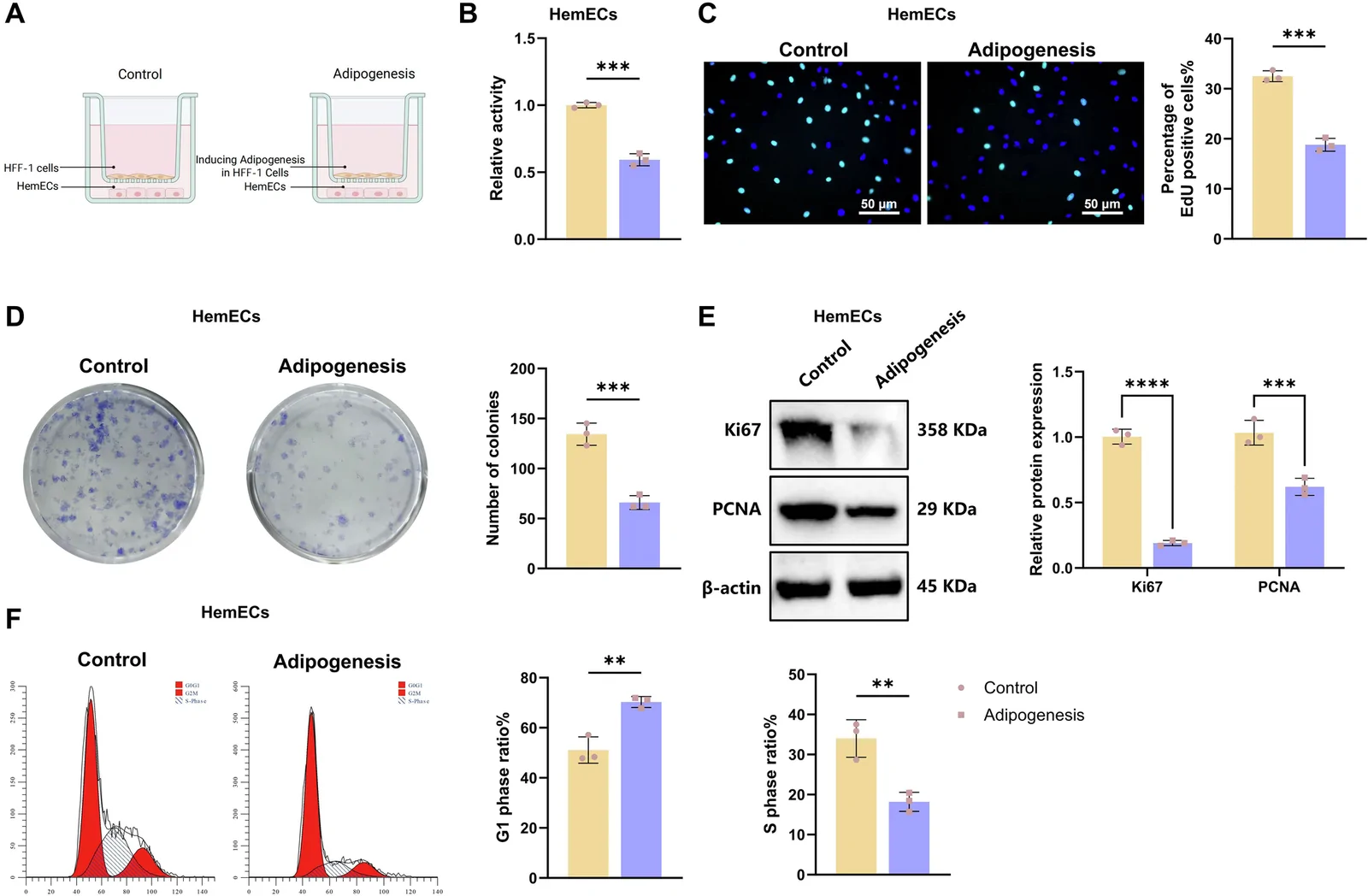
Effects of adipogenically differentiated FBs on HemEC viability and cell cycle progression. **A** Workflow of co-culture experiments with HFF-1 FBs and HemECs, created in Biorender. **B** CCK-8 assay evaluating changes in HemEC viability. **C** EdU staining assessing HemEC proliferative capacity (scale bar: 50 μm). **D** Colony formation assay determining clonogenic potential of HemECs (scale bar: 50 μm). **E** Western blot analysis of Ki67 and PCNA protein expression in HemECs. **F** Flow cytometry assessing HemEC cell cycle distribution. All experiments were performed in triplicate. ** indicates *p* < 0.01, *** indicates *p* < 0.001, **** indicates *p* < 0.0001 compared between groups.

A Transwell co-culture system was established with adipogenically differentiated HFF-1 FBs and HemECs to explore the impact of FB phenotypic changes on EC functional states (Fig. 2A). The cell counting kit-8 (CCK-8) assay revealed that, compared to the Control group, cell viability in the Adipogenesis group was significantly reduced, indicating that FBs post-adipogenic differentiation can continuously suppress HemEC activity (Fig. 2B). 5-Ethynyl-2′-deoxyuridine (EdU) staining demonstrated a significant decrease in the proportion of EdU-positive HemECs in the Adipogenesis group (Fig. 2C), and colony formation assays further confirmed a marked reduction in their clonogenic capacity (Fig. 2D). Western blot analysis suggested that proliferation-related proteins Ki67 and PCNA were significantly downregulated in Adipogenesis group ECs (Fig. 2E). Additionally, flow cytometry analysis showed a significant increase in the proportion of HemECs in the G1 phase and a decrease in the S phase, indicating cell cycle arrest (Fig. 2F).

**Fig. 3:**
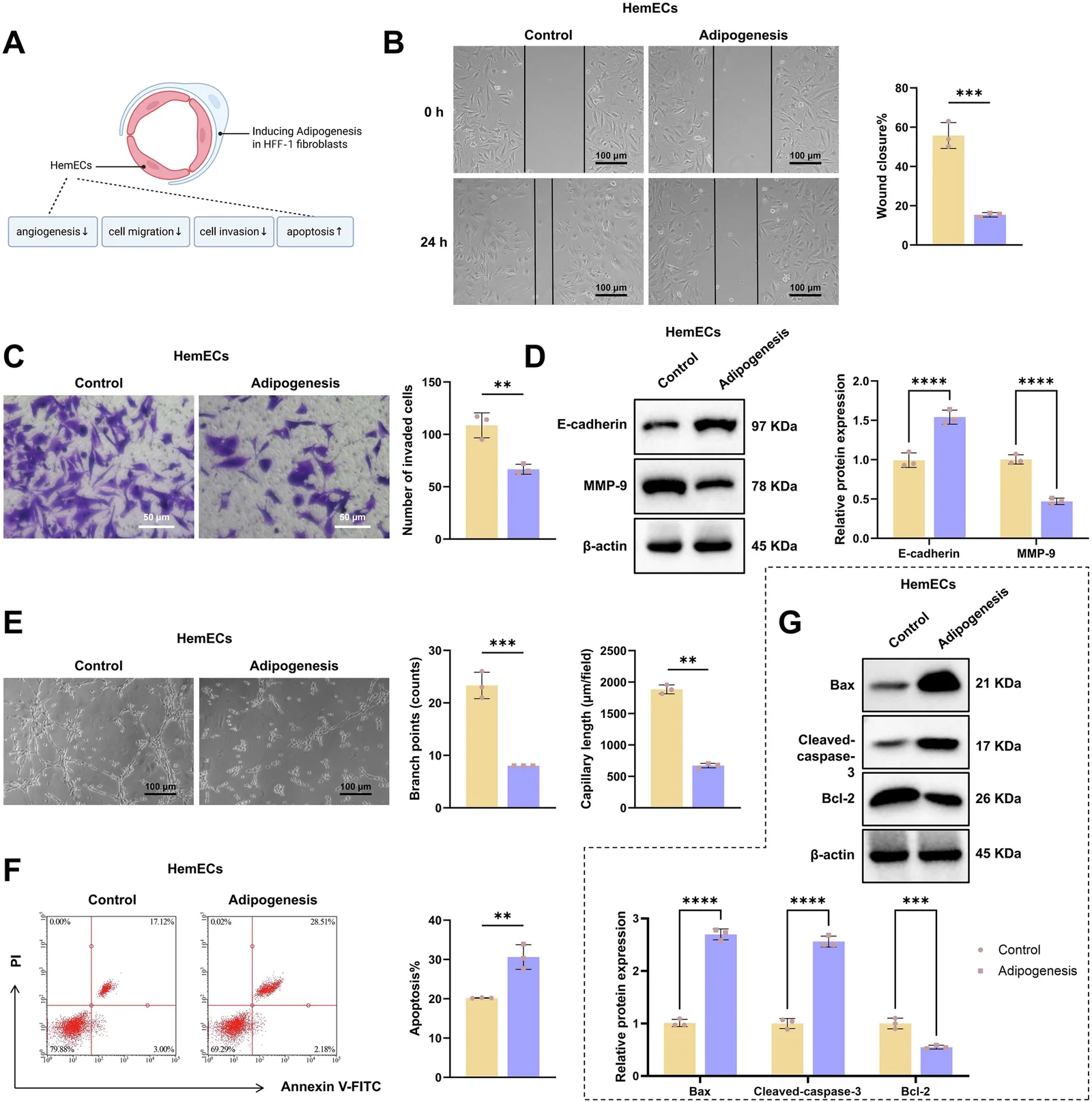
Effects of adipogenically differentiated FBs on HemEC migration, invasion, tube formation, and apoptosis. **A** Schematic illustration of the interactions between HemECs and adipogenically differentiated HFF-1 FBs, created in Biorender. **B** Wound healing assay assessing HemEC migratory ability (scale bar: 100 μm). **C** Transwell assay evaluating HemEC invasive capacity (scale bar: 50 μm). **D** Western blot analysis of E-cadherin and MMP-9 expression in HemECs. **E** Tube formation assay assessing capillary-like structure formation by HemECs (scale bar: 100 μm). **F** Flow cytometry quantification of HemEC apoptosis. **G** Western blot analysis of Bcl-2, Bax, and cleaved caspase-3 protein expression in HemECs. All experiments were performed in triplicate. ** indicates *p* < 0.01, ***p < 0.001, ****p < 0.0001 compared between groups.

Further assessments of HemEC migration, invasion, and angiogenesis were conducted (Fig. 3A). The wound healing assay indicated that the scratch closure rate was significantly lower in the Adipogenesis group than in the Control group, suggesting inhibited migration of HemECs (Fig. 3B). Transwell invasion assays showed a marked reduction in the number of transmigrated HemECs in the Adipogenesis group, indicating a significant decrease in invasive capacity (Fig. 3C). Western blot analysis revealed that E-cadherin expression was significantly upregulated while MMP-9 expression was downregulated in the Adipogenesis group HemECs (Fig. 3D), consistent with inhibited cell motility. Tube formation assays demonstrated a significant reduction in the number and length of tubular structures formed by HemECs in the Adipogenesis group (Fig. 3E). Flow cytometry analysis showed significantly increased early and late apoptosis in Adipogenesis group ECs (Fig. 3F), with Western blot validation showing significantly upregulated apoptosis-related proteins Bax and Cleaved-caspase-3 and downregulated Bcl-2 expression (Fig. 3G).

Together, these results demonstrate that adipogenic differentiation of fibroblasts markedly suppresses HemEC proliferation, migration, and angiogenic capacity while promoting apoptosis.

### Transcriptomic analysis identifies PPARγ as a key regulator linking fibroblast adipogenesis to hippo signaling in HemECs

**Fig. 4:**
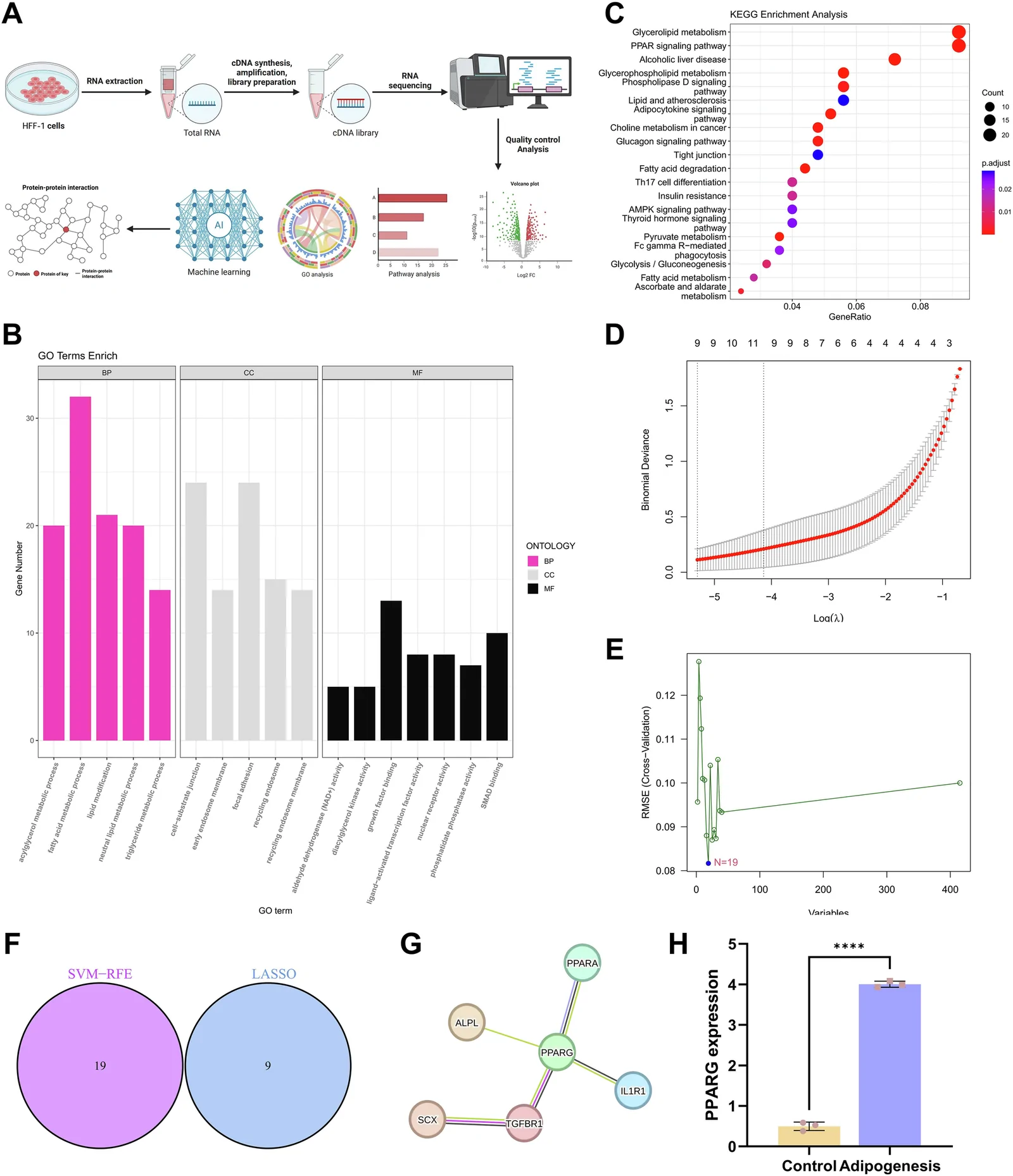
Transcriptomic analysis workflow and functional enrichment analysis of HFF-1 cells. **A** Workflow of transcriptomic analysis of HFF-1 cells, created in Biorender. **B** Gene Ontology (GO) enrichment analysis of differentially expressed genes (DEGs), including biological process (BP), cellular component (CC), and molecular function (MF) categories. **C** KEGG pathway enrichment analysis of signaling pathways associated with DEGs. **D** LASSO regression analysis for key gene selection. **E** SVM-RFE analysis for key gene selection. **F** Venn diagram showing the union of candidate genes identified by LASSO regression and SVM-RFE analyses. **G** Construction of the protein-protein interaction (PPI) network of differentially expressed genes and identification of hub genes; nodes represent proteins, and edges indicate PPIs, with thicker lines denoting higher confidence. **H** Bar chart showing the expression level of the hub gene PPARG. Control group, *n* = 3; Adipogenesis group, *n* = 3.

To explore the molecular mechanisms underlying the inhibitory effect of adipogenic fibroblasts on endothelial cells, we performed transcriptomic analyses of both fibroblasts and HemECs in the co-culture system. To elucidate the molecular basis by which adipogenically differentiated FBs influence ECs, we first performed transcriptomic sequencing of HFF-1 cells within the co-culture system (Fig. 4A). High-throughput sequencing was performed on three biological replicates from both the Control and Adipogenesis groups, revealing distinct transcriptional profiles as demonstrated by principal component analysis (PCA) (Fig. S4A). Differential expression analysis identified 415 significantly DEGs, with 204 upregulated and 211 downregulated (Fig. S4B, C). Subsequently, functional enrichment analyses were performed on these differentially expressed genes. Gene Ontology (GO) enrichment analysis showed that the differentially expressed genes were significantly enriched in biological process (BP) terms such as fatty acid metabolic process, cellular component (CC) terms including cell-substrate junction, and molecular function (MF) terms such as growth factor binding (Fig. 4B). Kyoto Encyclopedia of Genes and Genomes (KEGG) pathway enrichment analysis further highlighted significant enrichment in the Glycerolipid metabolism, PPAR signaling pathway, Glycerophospholipid metabolism, and Phospholipase D signaling pathway (Fig. 4C).

To further identify key differentially expressed genes (DEGs) with diagnostic or predictive potential, feature gene selection was performed using least absolute shrinkage and selection operator (LASSO) regression and support vector machine-recursive feature elimination (SVM-RFE) methods. These methods respectively identified 9 and 19 candidate key genes (Fig. 4D, E). The union of the gene sets obtained from both approaches yielded a total of 28 candidate key genes for subsequent analyses (Fig. 4F). Protein-protein interaction (PPI) analysis of these candidate genes revealed that PPARG occupied a central position within the constructed network, suggesting that it may represent a key regulatory hub in fibroblast adipogenic differentiation (Fig. 4G). Furthermore, examination of PPARG expression in the transcriptomic dataset demonstrated that, compared with the Control group, PPARG expression was significantly upregulated in the Adipogenesis group (Fig. 4H).

**Fig. 5:**
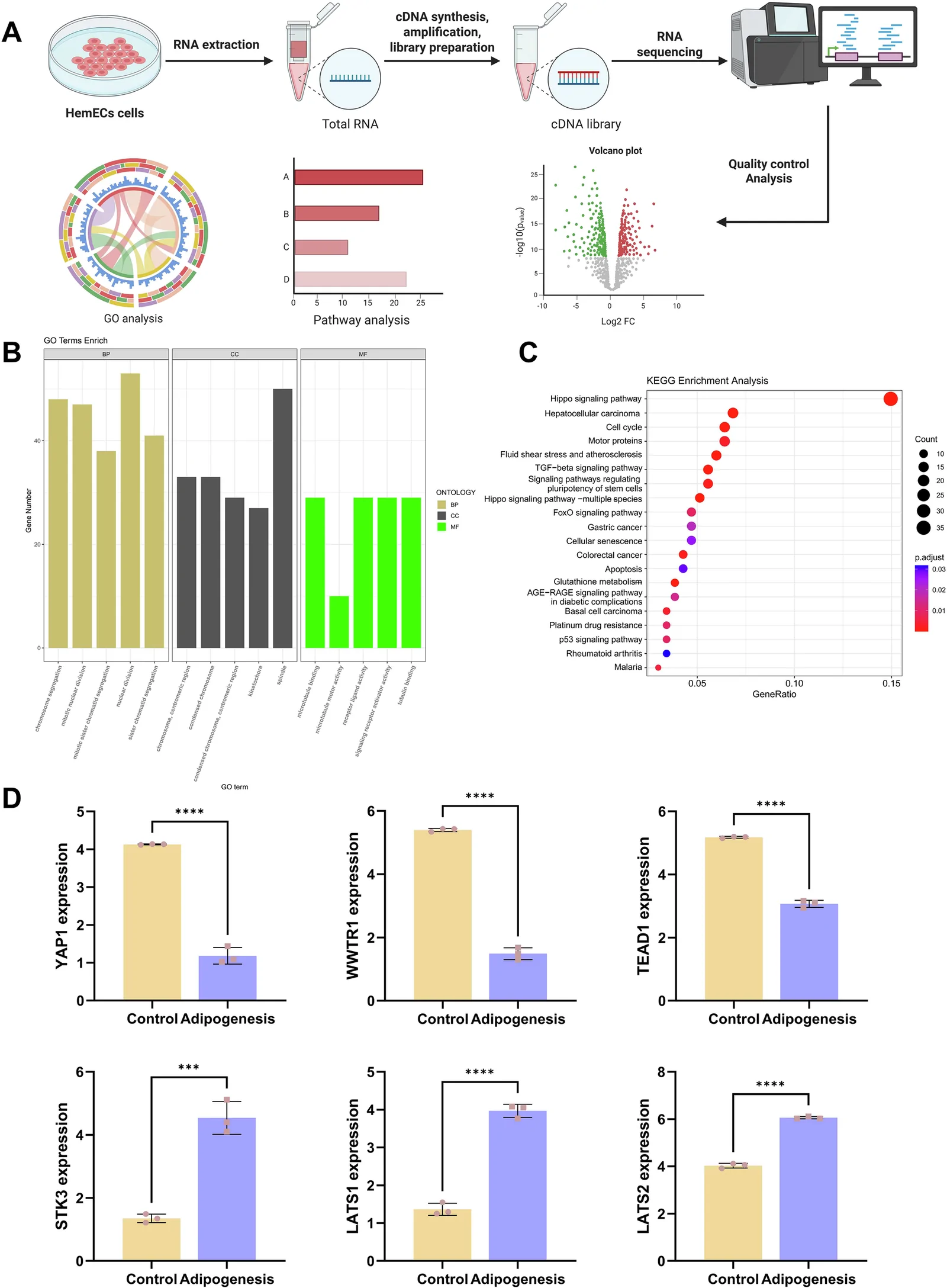
Transcriptomic analysis workflow and pathway enrichment analysis of HemECs. **A** Workflow of transcriptomic analysis of HemECs, created in Biorender. **B** GO enrichment analysis of biological processes associated with DEGs. **C** KEGG enrichment analysis of signaling pathways enriched by DEGs. **D** Expression levels of Hippo pathway core factors and upstream/downstream regulators. Control group, *n* = 3; Adipogenesis group, *n* = 3. *** indicates *p* < 0.001, **** *p* < 0.0001 compared between groups.

To further explore the impact of FB adipogenic differentiation on HemECs, RNA-seq was conducted on HemECs from the co-culture system (Fig. 5A). PCA analysis similarly demonstrated significant separation between the Control and Adipogenesis groups at the gene expression level (Fig. S4D). Differential expression analysis identified 447 significantly DEGs, with 200 upregulated and 247 downregulated (Fig. S4E, F). GO enrichment analysis revealed significant enrichment in the categories of nuclear division, spindle, and signaling receptor activator activity (Fig. 5B). KEGG pathway enrichment analysis highlighted significant enrichment in the Hippo signaling pathway, the Cell cycle, and the Hippo signaling pathway - multiple species (Fig. 5C). Further analysis showed that the expression levels of YAP (*YAP1*), TAZ (*WWTR1*), and their downstream transcription factor TEAD (*TEAD1*) were notably downregulated in the Adipogenesis group, while the upstream regulators of the Hippo Pathway, MST1/2 (STK3) and LATS1/2 (*LATS1*, *LATS2*), were significantly upregulated (Fig. 5D), suggesting activation of this pathway and potential negative regulation of EC functions.

These findings suggest that PPARγ may function as a key regulator of fibroblast adipogenic differentiation and that Hippo signaling is likely involved in mediating the downstream effects on endothelial cells.

### PPARγ-driven fibroblast adipogenesis activates Hippo signaling and suppresses HemEC function

**Fig. 6:**
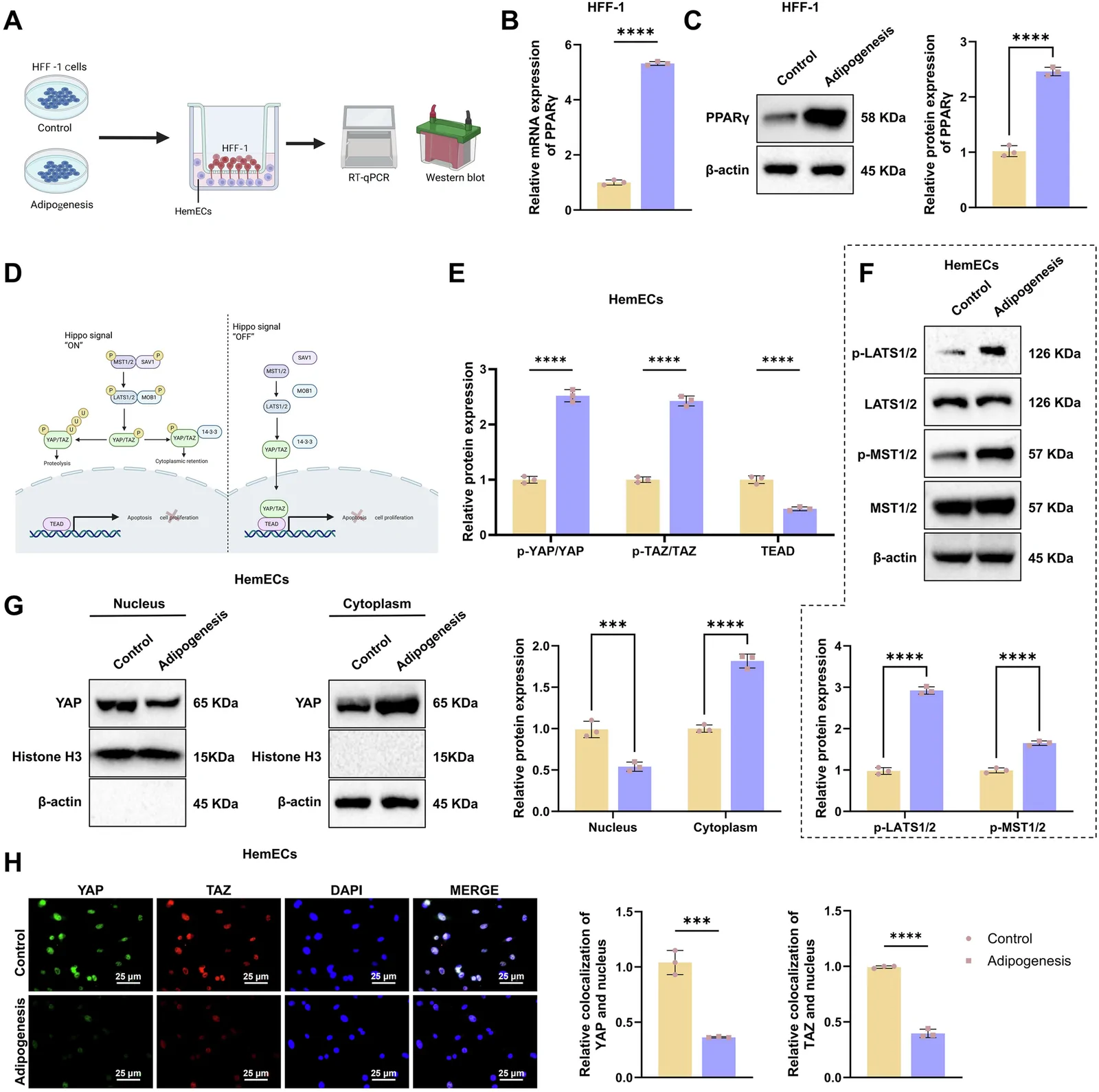
Detection of PPARγ expression and Hippo-YAP/TAZ pathway activity. **A** Schematic of the Transwell co-culture system, created in Biorender. **B** RT-qPCR analysis of PPARγ mRNA expression in HFF-1 cells. **C** Western blot analysis of PPARγ protein expression in HFF-1 cells. **D** Diagram of the Hippo signaling axis, created in Biorender. **E** Western blot analysis of YAP, p-YAP, TAZ, p-TAZ, and TEAD protein expression in HemECs. **F** Western blot analysis of MST1/2, p-MST1/2, LATS1/2, and p-LATS1/2 protein expression in HemECs. **G** Western blot analysis of YAP expression in nuclear and cytoplasmic fractions of HemECs. **H** IF co-staining showing subcellular localization of YAP and TAZ in HemECs (scale bar: 25 μm). All experiments were repeated three times. *** indicates *p* < 0.001, ****p < 0.0001 compared between groups.

To validate the regulatory role of PPARγ and its relationship with Hippo signaling, we performed mechanistic experiments using PPARG knockdown in fibroblasts and examined the resulting changes in endothelial cell behavior. To validate the results of transcriptome analysis and further elucidate the pivotal regulatory role of PPARγ and the Hippo-YAP/TAZ signaling pathway in the interaction between FB and EC, a co-culture model of HFF-1 FB and HemECs EC was established, followed by mechanistic experiments (Fig. 6A). Reverse transcription quantitative polymerase chain reaction (RT-qPCR) and Western blot analyses revealed a significant upregulation of both mRNA and protein levels of PPARγ in HFF-1 cells of the Adipogenesis group compared to the Control group (Fig. 6B, C), suggesting that PPARγ may play a crucial regulatory role in adipogenic differentiation.

To investigate the impact of altered PPARγ expression on the Hippo pathway in EC (Fig. 6D), the expression changes of key factors [35, 36] in HemECs were examined. The findings indicated a marked increase in the phosphorylation levels of YAP and TAZ (p-YAP, p-TAZ) in the Adipogenesis group compared to the Control group, with a concomitant significant decrease in the expression of the downstream transcription factor TEAD protein (Fig. 6E). Additionally, the phosphorylated forms of upstream regulatory factors MST1/2 and LATS1/2 (p-MST1/2 and p-LATS1/2) were significantly elevated in the Adipogenesis group (Fig. 6F), indicating an activation trend of the Hippo pathway. Using a nuclear and cytoplasmic extraction kit, the subcellular localization of YAP was analyzed, showing a notable decrease in nuclear YAP and an increase in cytoplasmic YAP in the Adipogenesis group (Fig. 6G). Further IF staining revealed significantly weakened nuclear fluorescence signals of both YAP and TAZ in the Adipogenesis group (Fig. 6H).

**Fig. 7:**
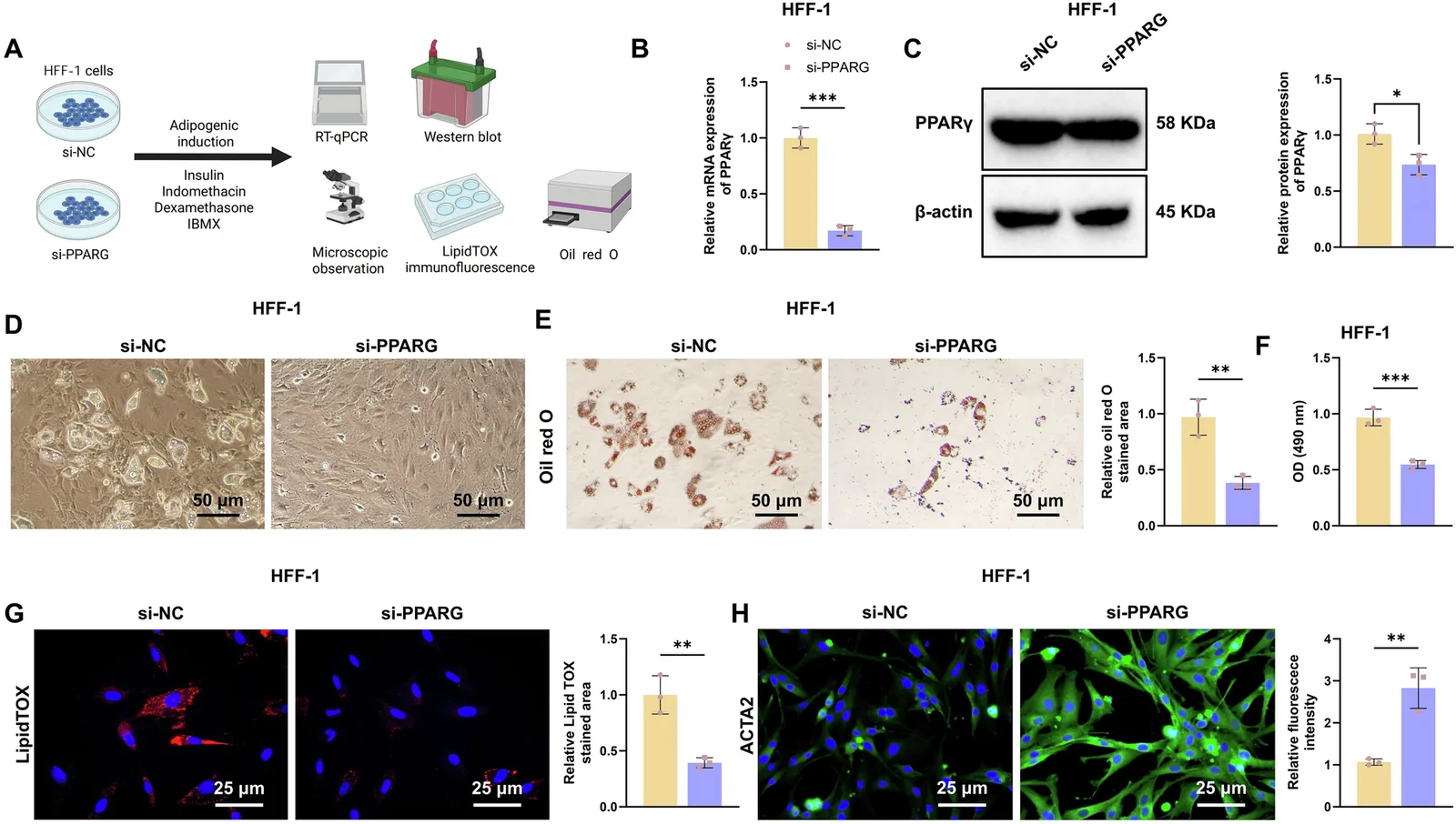
Assessment of adipogenic differentiation capacity in HFF-1 cells following PPARG silencing. **A** Workflow of adipogenic differentiation experiments in PPARG-silenced HFF-1 cells, created in Biorender. **B** RT-qPCR analysis of PPARG mRNA expression in HFF-1 cells. **C** Western blot analysis of PPARγ protein expression in HFF-1 cells. **D** Microscopic observation of HFF-1 cell morphology (scale bar: 50 μm). **E**, **F** ORO staining detecting lipid droplet formation in HFF-1 cells, with lipid accumulation quantified by OD measurement. **G** LipidTOX staining showing lipid droplet distribution in HFF-1 cells (scale bar: 25 μm). **H** IF staining assessing ACTA2 filament structures in HFF-1 cells (scale bar: 25 μm). All experiments were performed in triplicate. * indicates *p* < 0.05, ** indicates *p* < 0.01, *** *p* < 0.001 compared between groups.

**Fig. 8:**
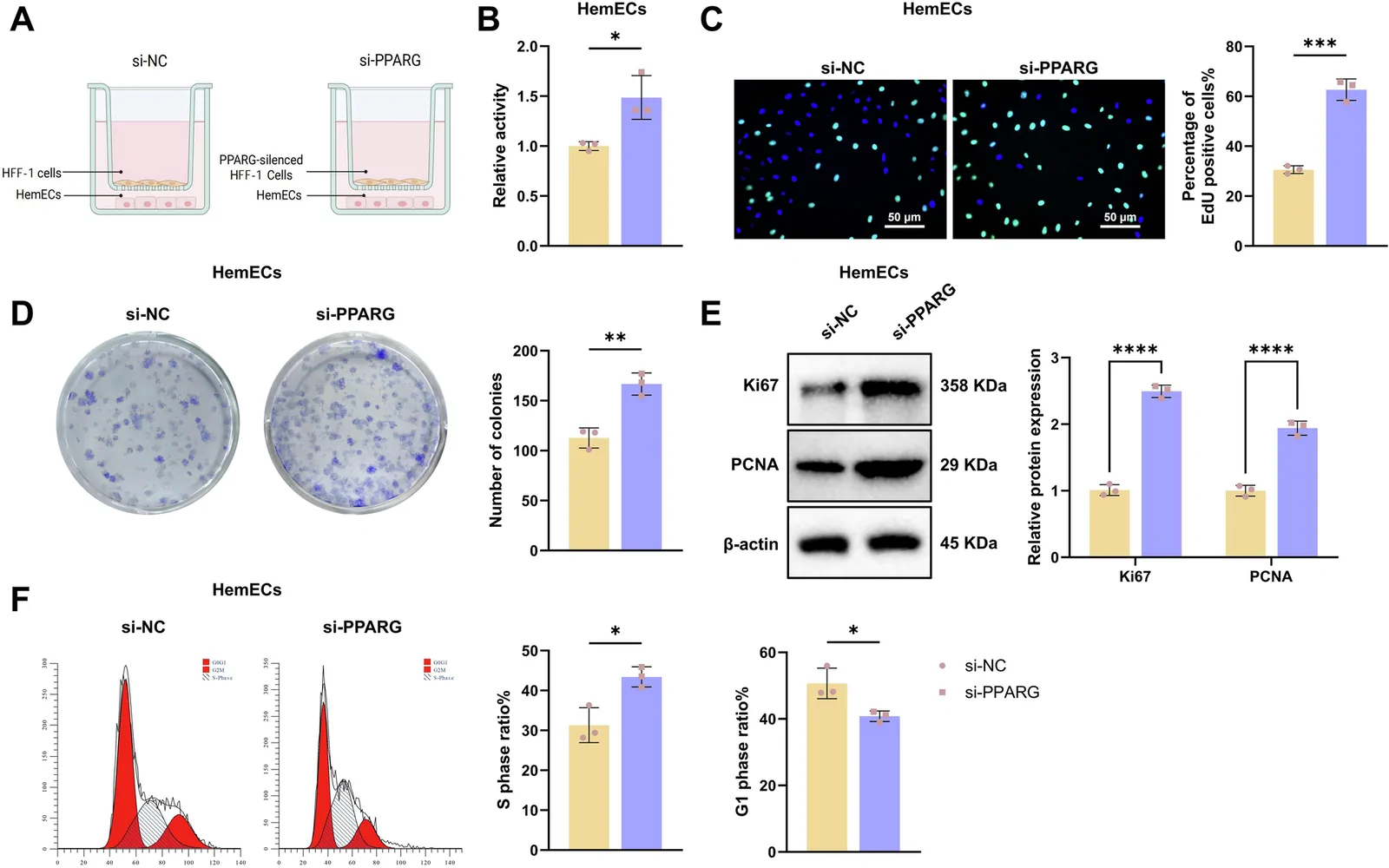
Effects of PPARG silencing on HemEC proliferation and cell cycle. **A** Workflow of the Transwell co-culture system, created in Biorender. **B** CCK-8 assay assessing HemEC viability. **C** EdU staining evaluating HemEC proliferative capacity (scale bar: 50 μm). **D** Colony formation assay determining HemEC clonogenic capacity. **E** Western blot analysis of Ki67 and PCNA protein expression in HemECs. **F** Flow cytometry assessing HemEC cell cycle distribution. All experiments were performed in triplicate. * indicates *p* < 0.05, ** *p* < 0.01, ***p < 0.001, ****p < 0.0001 compared between groups.

**Fig. 9:**
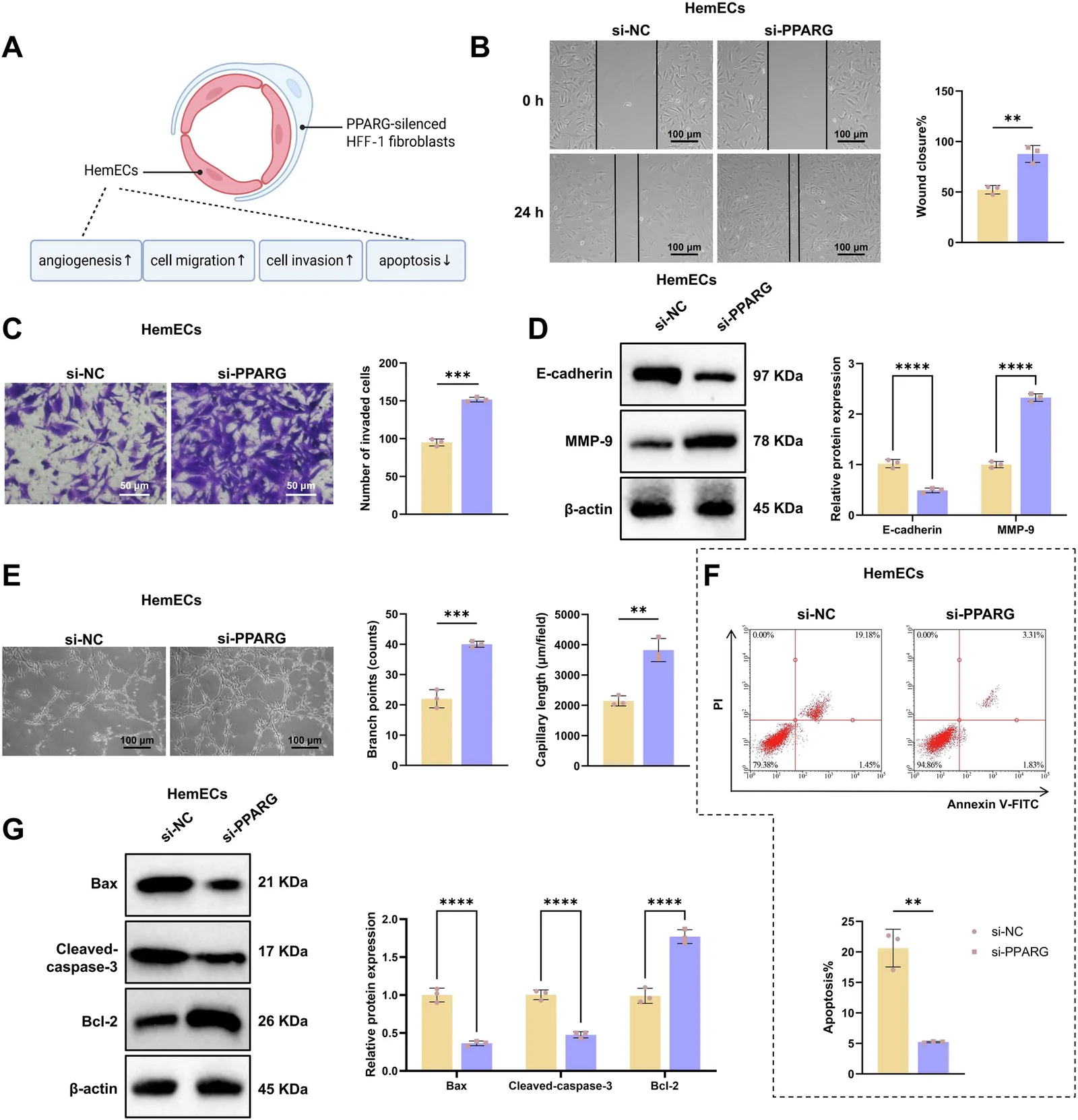
Effects of PPARG silencing on HemEC migration, invasion, tube formation, and apoptosis. **A** Schematic illustration of the interactions between HemECs and PPARG-silenced HFF-1 FBs, created in Biorender. **B** Wound-healing assay assessing HemEC migratory capacity (scale bar: 100 μm). **C** Transwell assay evaluating HemEC invasive ability (scale bar:50 μm). **D** Western blot analysis of E-cadherin and MMP-9 protein expression in HemECs. **E** Tube formation assay examining capillary-like structure formation by HemECs (scale bar: 100 μm). **F** Flow cytometry analysis of HemEC apoptosis. **G** Western blot analysis of Bcl-2, Bax, and cleaved caspase-3 protein expression in HemECs. All experiments were performed in triplicate. **p < 0.01, ***p < 0.001, ****p < 0.0001 compared between groups.

Next, to validate the necessity of PPARγ in this regulatory axis from an opposite perspective, we established a PPARG knockdown model using small interfering RNA (si-PPARG). As shown in Fig. 7A–C, efficient silencing of PPARG was achieved. In contrast to the phenotypes induced by PPARγ activation, PPARG knockdown effectively blocked adipogenic differentiation in HFF-1 cells, as evidenced by a marked reduction in lipid droplet accumulation (Fig. 7D–G) and the restoration of ACTA2 filamentous structures (Fig. 7H). We then investigated the impact of impaired PPARγ function in fibroblasts on endothelial cells within the co-culture system. Strikingly, opposite to the inhibitory effects observed in the Adipogenesis group, co-culture with si-PPARG fibroblasts significantly reversed the suppressed endothelial phenotypes. Specifically, HemECs exhibited enhanced cell viability and proliferative capacity, as confirmed by CCK-8 assays, EdU incorporation, colony formation assays, and the expression of proliferation markers Ki67 and PCNA (Fig. 8B–F). In addition, endothelial cell migration, invasion, and tube formation abilities were markedly increased, whereas apoptosis levels were significantly reduced (Fig. 9A–G). Importantly, at the molecular level, PPARG knockdown reversed the adipogenesis-induced activation of the Hippo signaling pathway in HemECs (data not shown), thereby mechanistically establishing PPARγ as an upstream core regulator within this signaling axis. These results confirm that PPARγ plays a central role in fibroblast-mediated regulation of endothelial cell function through activation of the Hippo signaling pathway.

### Activation of Hippo signaling suppresses HemEC function, whereas pharmacological inhibition reverses this Effect

**Fig. 10:**
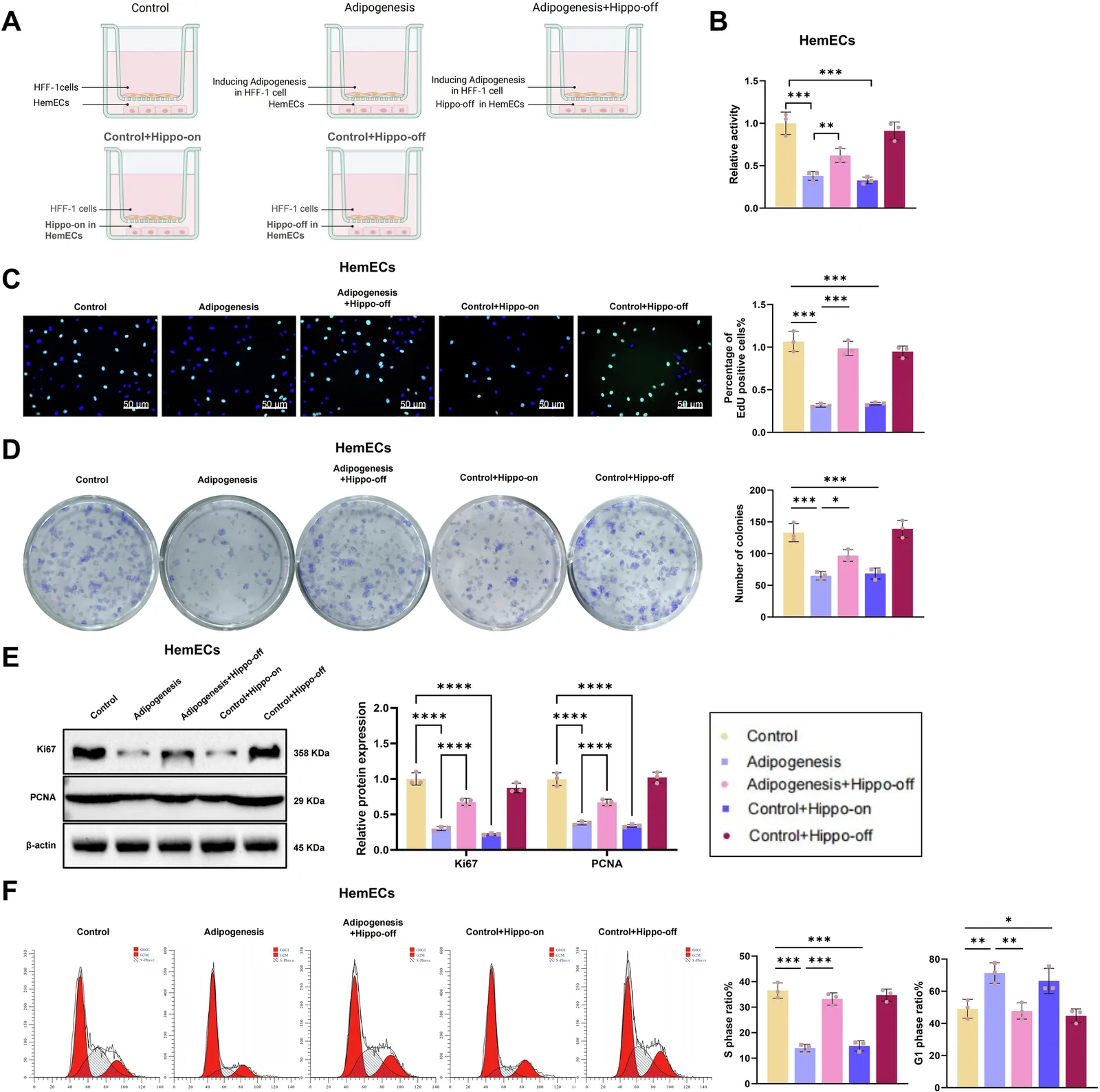
Effects of Hippo pathway inhibition on HemEC viability and proliferation. **A** Workflow of the Transwell co-culture experiment, created in Biorender. **B** CCK-8 assay assessing changes in HemEC viability. **C** EdU staining evaluating HemEC proliferative capacity (scale bar: 50 μm). **D** Colony formation assay assessing clonogenic potential of HemECs. **E** Western blot analysis of Ki67 and PCNA protein expression in HemECs. **F** Flow cytometry assessing HemEC cell cycle distribution. All experiments were performed in triplicate. * indicates *p* < 0.05, ** *p* < 0.01, *** *p* < 0.001, **** *p* < 0.0001 compared between groups.

To further determine whether Hippo signaling mediates the inhibitory effects of adipogenic fibroblasts on HemEC function, we examined the impact of both Hippo pathway activation and pharmacological inhibition in the co-culture system. Three co-culture systems were established to investigate the role of the Hippo Pathway in regulating the function of HemECs by adipogenic differentiation of FBs. The groups included: the Control group, the Adipogenesis group, the Control+Hippo-on group, the Control+Hippo-off group, and the Adipogenesis+Hippo-off group treated with the Hippo Pathway inhibitor XMU-MP-1 (Fig. 10A). CCK-8 assays indicated a significant decrease in EC viability in the Adipogenesis and Control+Hippo-on groups compared to the Control group, while the Adipogenesis+Hippo-off group showed a marked recovery and increase in cell viability compared to the Adipogenesis group (Fig. 10B). EdU staining assays revealed a significant reduction in EdU-positive cells in the Adipogenesis and Control+Hippo-on groups, whereas inhibition of the Hippo Pathway significantly increased their number (Fig. 10C). Colony formation assays also demonstrated the restoration of clonogenic capacity in the Adipogenesis+Hippo-off group (Fig. 10D). Western blot analysis showed downregulation of Ki67 and PCNA protein expression in the Adipogenesis and Control+Hippo-on groups, which was significantly upregulated following Hippo Pathway inhibition (Fig. 10E). Flow cytometry indicated an increase in the G1-phase cell population and a decrease in the S-phase in the Adipogenesis and Control+Hippo-on groups, with the opposite trend observed in the Adipogenesis+Hippo-off group (Fig. 10F). In addition, in the above experiments, no statistically significant differences were observed between the Control+Hippo-off group and the Control group. These findings indicate that the inhibitor XMU-MP-1 specifically reversed the inhibitory effects induced by adipogenic differentiation, rather than independently affecting the basal functions of HemECs.

**Fig. 11:**
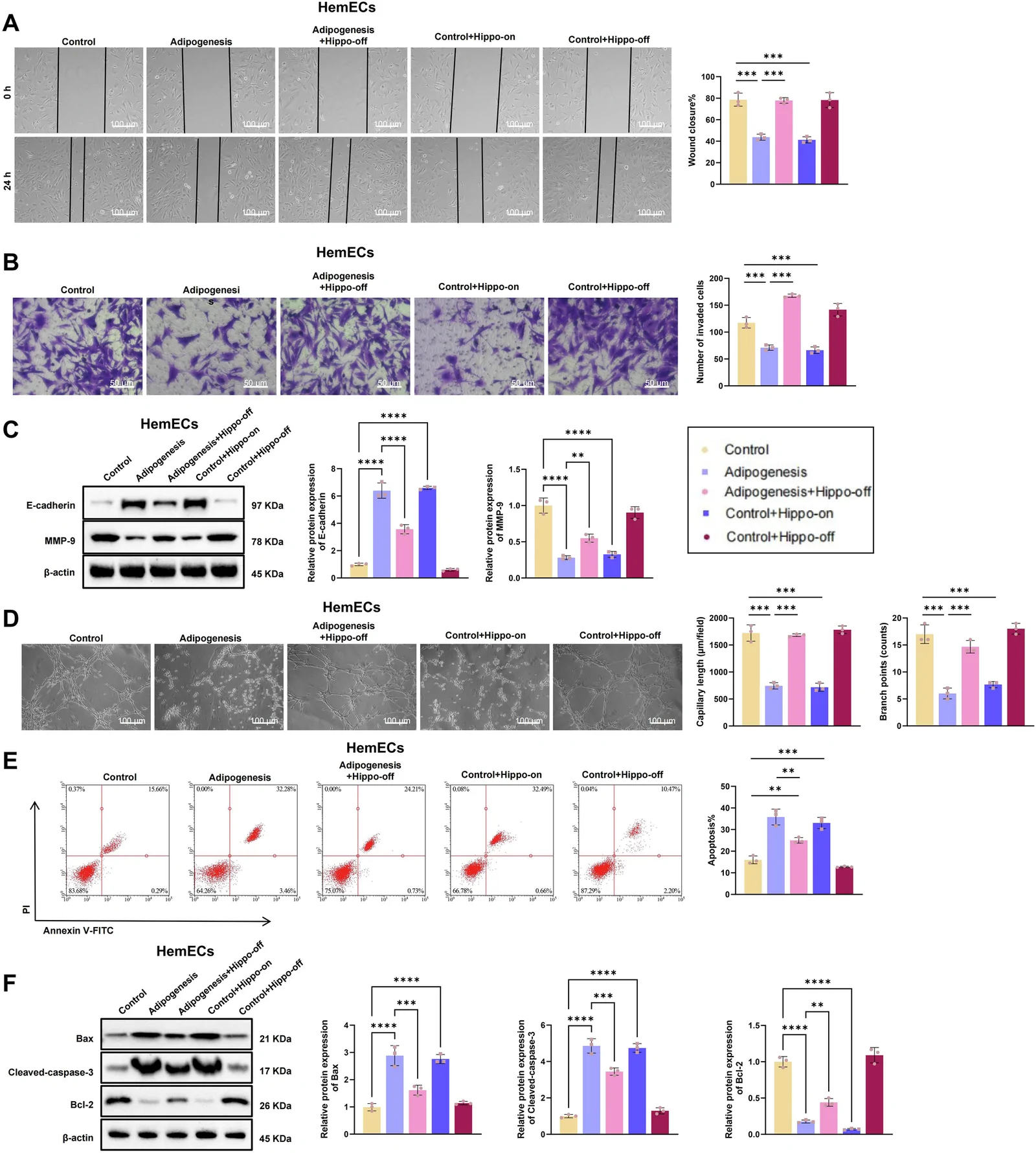
Effects of Hippo pathway inhibition on HemEC migration, invasion, tube formation, and apoptosis. **A** Wound-healing assay assessing HemEC migratory capacity (scale bar: 100 μm). **B** Transwell assay evaluating HemEC invasive ability (scale bar: 50 μm). **C** Western blot analysis of E-cadherin and MMP-9 protein expression in HemECs. **D** Tube formation assay assessing angiogenic capacity of HemECs (scale bar: 100 μm). **E** Flow cytometry analysis of HemEC apoptosis. **F** Western blot analysis of Bax, Bcl-2, and cleaved caspase-3 protein expression in HemECs. All experiments were performed in triplicate. * indicates *p* < 0.05, ** *p* < 0.01, *** *p* < 0.001, **** *p* < 0.0001 compared between groups.

Further assessments of EC migration, invasion, and tube formation capabilities were conducted. Wound healing assays demonstrated slower scratch closure in the Adipogenesis and Control+Hippo-on groups compared to the Control group, while the Hippo Pathway inhibition group exhibited significantly enhanced migration ability (Fig. 11A). Transwell invasion assays indicated a decrease in transmembrane cell numbers in the Adipogenesis and Control+Hippo-on groups, with a significant increase in the Adipogenesis+Hippo-off group (Fig. 11B). Western blot results showed a significant upregulation of E-cadherin and a downregulation of MMP-9 expression in the Adipogenesis and Control+Hippo-on groups, which were reversed by Hippo Pathway inhibition (Fig. 11C). Tube formation assays revealed a reduction in tubular structures formed by ECs in the Adipogenesis group, which was counteracted by the addition of XMU-MP-1, enhancing tube-forming ability (Fig. 11D). Flow cytometry results showed an increased apoptosis rate in the Adipogenesis and Control+Hippo-on groups, which was significantly reduced by Hippo Pathway inhibition (Fig. 11E). Western blot analysis further confirmed the upregulation of Bax and Cleaved-caspase-3 and the downregulation of Bcl-2 in the Adipogenesis and Control+Hippo-on groups, with opposite trends observed in the Adipogenesis+Hippo-off group (Fig. 11F). In addition, in the above experiments, no statistically significant differences were observed between the Control+Hippo-off group and the Control group. These findings indicate that the inhibitor XMU-MP-1 specifically reversed the inhibitory effects induced by adipogenic differentiation, rather than independently affecting the basal functions of HemECs.

Western blot analysis indicated that, compared to the Control group, the levels of phosphorylated YAP and TAZ (p-YAP, p-TAZ) were significantly elevated, and TEAD expression was reduced in the Adipogenesis and Control+Hippo-on groups. In contrast, these indicators were markedly reversed in the Adipogenesis+Hippo-off group, with decreased phosphorylation of YAP and TAZ and a significant upregulation of TEAD expression (Fig. S5A). Further analysis of upstream regulatory factors of the Hippo Pathway, MST1/2 and LATS1/2, was performed. Western blot results showed significant upregulation of p-MST1/2 and p-LATS1/2 expression levels in the Adipogenesis and Control+Hippo-on groups compared to the Control group, with a significant downregulation following the addition of the Hippo Pathway inhibitor (Fig. S5B). These findings suggest that adipogenic differentiation activates the Hippo Pathway, whereas XMU-MP-1 effectively inhibits its activity.

To verify the impact of pathway activity changes on YAP subcellular localization, nuclear-cytoplasmic fractionation was used to extract nuclear and cytoplasmic proteins from ECs, followed by Western blot analysis. The results indicated a significant reduction in nuclear YAP expression and a significant increase in cytoplasmic expression in the Adipogenesis and Control+Hippo-on groups compared to the Control group. The Adipogenesis+Hippo-off group exhibited a significant restoration of YAP nuclear localization (Fig. S5C). IF staining demonstrated that the nuclear fluorescence signals of YAP and TAZ were markedly weakened in the Adipogenesis and Control+Hippo-on groups, while XMU-MP-1 treatment significantly enhanced their nuclear localization (Fig. S5D).

Taken together, these results demonstrate that activation of Hippo signaling contributes to the suppression of HemEC function, whereas pharmacological inhibition of this pathway reverses these effects. These findings suggest that controlled activation of Hippo signaling may represent a potential therapeutic strategy for HEM.

## Discussion

In the present study, we identified fibroblast adipogenic differentiation as a critical microenvironmental regulator of hemangioma endothelial cell function. By integrating scRNA-seq analysis with transcriptomic profiling and functional experiments, we demonstrate that PPARγ-driven adipogenic differentiation of fibroblasts suppresses endothelial cell proliferation, migration, and angiogenic capacity while promoting apoptosis. Mechanistically, this effect is mediated through activation of the Hippo-YAP/TAZ signaling pathway in endothelial cells. These findings reveal a previously unrecognized stromal-endothelial regulatory axis that contributes to the suppression of HEM progression.

In recent years, the application of scRNA-seq technology has unveiled significant cellular heterogeneity within HEM tissues, encompassing functional disparities among EC subpopulations and diverse distribution patterns of immune cell groups [12]. Through reanalysis of scRNA-seq data, our study not only corroborates this notion but also identifies a high degree of heterogeneity in the distribution and activity levels of FBs within HEM tissues. More importantly, our CellChat communication analysis reveals intricate L-R interactions between FBs and ECs, starkly contrasting with previous research that focused solely on EC characteristics. Furthermore, in vitro experiments validate the significant impact of FBs in various differentiation states on EC functionality, offering new evidence for understanding the complexity of multicellular interactions within HEM tissues.

PPARγ has long been recognized as a pivotal regulator of lipid metabolism and adipogenic differentiation. Previous studies have documented the role of PPARγ agonists in improving vascular function or regulating metabolism in conditions such as atherosclerosis, diabetes, and certain tumors [37, 38]. However, research on PPARγ in HEM remains relatively sparse. Our study reveals that the PPARG gene is significantly upregulated in FBs during adipogenic differentiation, and this change is closely associated with a decline in EC function, thereby addressing a gap in the current research landscape. Unlike previous investigations that primarily explored the role of PPARγ in metabolic disorders or malignant tumors, our study is the first to link PPARγ with benign vascular tumors. Notably, we found that the core effect of PPARγ lies in intercellular interactions rather than autonomous regulation within individual cells, which contrasts with the traditional focus of earlier studies. Therefore, PPARγ should be viewed not merely as a factor in metabolic regulation but as a crucial node within the microenvironmental signaling network.

In recent years, the Hippo-YAP/TAZ pathway has emerged as a focal point in angiogenesis and tumor research [21]. Previous studies have demonstrated that when activated, YAP/TAZ can enhance the proliferation of ECs and promote angiogenesis, contributing to the progression of various vascular-related diseases [39, 40]. From a mechanistic perspective, the Hippo pathway primarily exerts its negative regulatory function through a mechanism of “spatial segregation.” Upon activation of the upstream kinase cascade (MST1/2–LATS1/2), the effector proteins YAP and TAZ become phosphorylated and are subsequently captured by 14-3-3 proteins and anchored in the cytoplasm. This cytoplasmic sequestration effectively prevents YAP/TAZ from translocating into the nucleus and interacting with TEAD transcription factors, thereby shutting down the transcriptional program of downstream pro-proliferative genes both physically and functionally [20, 41, 42]. Our findings are consistent with these established concepts, while also providing a novel perspective. Specifically, we demonstrate that adipogenic differentiation of fibroblasts activates the Hippo signaling cascade in endothelial cells through a PPARγ-mediated mechanism, resulting in increased YAP/TAZ phosphorylation, reduced nuclear localization, and subsequent suppression of HemEC function. In contrast to previous studies that primarily attributed HEM development to intrinsic signaling abnormalities within endothelial cells, our work proposes a new model in which the metabolic state of surrounding stromal cells indirectly modulates endothelial cell behavior via intercellular signaling pathways. This discovery broadens the current understanding of the role of the Hippo-YAP/TAZ pathway in pathological vascular proliferation, highlighting that its regulation is shaped not only by cell-intrinsic mechanisms but also by cross-cellular cues within the microenvironment.

However, how activation of nuclear PPARγ in fibroblasts crosses cellular boundaries to suppress YAP/TAZ activity in neighboring endothelial cells remains unclear. To address this “black box,” we propose a dual metabolic–mechanical regulatory hypothesis based on existing literature and our own data. First, activation of PPARγ may remodel the microenvironmental secretory profile through paracrine mechanisms. Previous studies have shown that PPARγ agonists markedly suppress vascular endothelial growth factor (VEGF) secretion by fibroblasts [43], while VEGF is a well-established activator of YAP/TAZ signaling in endothelial cells [44]. In parallel, adipogenic differentiation is accompanied by a pronounced increase in adiponectin secretion. Recent evidence indicates that adiponectin promotes YAP/TAZ phosphorylation and degradation via the AMPK/SIRT1 axis [45]. Collectively, this “low-VEGF/high-adiponectin” microenvironment may constitute a chemical signaling milieu that suppresses endothelial YAP/TAZ activity. Second, from a biomechanical perspective, the transition of fibroblasts into lipid-laden adipocytes is associated with cytoskeletal reorganization and a reduction in ECM stiffness. Given that YAP/TAZ are canonical mechanosensors that respond dynamically to changes in matrix rigidity [46], we speculate that the PPARγ-induced “adipogenic soft matrix” deprives endothelial cells of the mechanical support provided by a stiff substrate, thereby promoting YAP/TAZ nuclear export and functional inactivation. The coordinated action of these chemical and physical cues provides a plausible explanation for the seemingly paradoxical cross-cellular connection between PPARγ activation and Hippo–YAP/TAZ pathway suppression, offering a mechanistic framework for intercellular regulation within the HEM microenvironment.

This study provides bidirectional and robust experimental evidence supporting a central role for the Hippo–YAP/TAZ pathway in regulating HemEC function. On the one hand, our gain-of-function experiments demonstrate that when fibroblasts undergo PPARγ-driven adipogenic differentiation, the resulting microenvironment is sufficient to activate the Hippo pathway in co-cultured endothelial cells, as evidenced by increased YAP/TAZ phosphorylation and reduced nuclear localization, ultimately leading to marked suppression of endothelial cell function. On the other hand, our loss-of-function experiments further show that pharmacological blockade of this pathway using the specific inhibitor XMU-MP-1 effectively reverses the endothelial dysfunction induced by the adipogenic microenvironment. This complementary “gain-and-loss” line of evidence not only confirms that Hippo pathway activation is a necessary event mediating endothelial functional suppression, but also aligns with the classical concept that Hippo pathway activity is inversely correlated with cellular proliferation [21]. Consequently, our conclusions avoid the limitations inherent to single-line evidence and strongly support the reliability of the Hippo–YAP/TAZ signaling axis as a potential therapeutic target in HEM.

Current clinical treatments for HEM primarily focus on pharmacotherapy, with propranolol being considered a significant breakthrough. Its main mechanisms of action include vasoconstriction, inhibition of EC proliferation, and induction of apoptosis [47, 48]. However, these treatment approaches mainly target ECs themselves and have limited regulatory effects on the surrounding microenvironment cells. Our study suggests that modulating the differentiation state of FBs can significantly influence the progression of HEM, thereby offering potential avenues for developing novel therapeutic strategies. As a key factor in adipogenic differentiation, PPARγ emerges as a potential therapeutic target. Based on the above bidirectional validation, this study demonstrates that modulation of Hippo pathway activity—whether indirectly through upstream PPARγ activation or directly via pharmacological intervention—can profoundly influence the fate of HemECs. These findings suggest that future therapeutic strategies may offer greater flexibility: on the one hand, mimicking an adipogenic microenvironment to activate the Hippo pathway (for example, through the use of PPARγ agonists) could be employed to promote tumor regression; on the other hand, directly targeting key nodes of the Hippo pathway may enable precise control of endothelial cell behavior. Compared with current treatment strategies that primarily focus on β-adrenergic receptor blockade in endothelial cells, this novel approach targeting both the microenvironment and core signaling pathways provides an additional therapeutic dimension for refractory HEM. Importantly, evidence supporting the Hippo pathway as a therapeutic target is derived not only from loss-of-function experiments, but also from pivotal gain-of-function studies. For instance, experimental overexpression of MST1 in human umbilical vein endothelial cells (HUVECs) via adenoviral vectors was sufficient to disrupt tubular network formation on Matrigel and markedly inhibit cell migration [49]. In addition, pharmacological reactivation of Hippo signaling using verteporfin elicited pronounced anti-angiogenic effects [50]. Of particular clinical relevance, in models of disturbed flow–induced endothelial inflammation, reactivation of MST1 successfully restored the expression of inflammatory molecules such as VCAM-1, demonstrating the therapeutic potential of Hippo pathway activation in correcting pathological endothelial states.

In recent years, the tumor microenvironment has garnered significant attention in malignant tumor research as a pivotal factor regulating tumorigenesis and progression [51, 52]. However, the role of the microenvironment in benign HEM research is often overlooked. This study demonstrates that the progression of HEM also relies on the state of FBs within the microenvironment and their interaction with ECs. Unlike studies on malignant tumors, which emphasize an immunosuppressive microenvironment, our research reveals that changes in the metabolic state of FBs inhibit EC function. This finding suggests that while there are differences in microenvironmental regulatory mechanisms between benign and malignant tumors, there are also potential commonalities. By further expanding the scope of microenvironmental studies, we can not only gain a better understanding of the biological characteristics of HEM but also potentially identify molecular regulatory networks shared across different tumor types, providing insights for therapeutic strategies.

This study employs a multidimensional research strategy that integrates scRNA-seq, RNA-seq, and in vitro functional experiments, enhancing the reliability and comprehensiveness of its conclusions. Compared to previous studies that relied solely on histological observations or single experimental methods, this research provides a more holistic understanding of intercellular interactions and molecular mechanisms. However, certain limitations must be acknowledged. Firstly, the primary validations in this study are conducted in vitro, lacking further support from in vivo animal models and clinical samples, which may affect the generalizability and translational potential of the findings. Secondly, the regulation of PPARγ and the Hippo Pathway is tissue-specific; whether these findings are applicable to other types of HEM or different pathological conditions requires further investigation. Additionally, the safety and long-term efficacy of targeting PPARγ or the Hippo Pathway with drugs need clinical validation. Therefore, although the results are promising, they should be cautiously applied on a broader scale.

This study establishes a co-culture model of adipogenically differentiated fibroblasts and HemECs and integrates transcriptomic analyses with functional assays to elucidate the critical role of PPARγ in fibroblast adipogenic differentiation. Our findings demonstrate that PPARγ-driven adipogenic remodeling of fibroblasts suppresses HemEC proliferation, migration, invasion, and angiogenic capacity while promoting apoptosis through activation of the Hippo-YAP/TAZ signaling pathway. These results identify the PPARγ–Hippo axis as a key regulatory mechanism linking fibroblast phenotypic remodeling to endothelial dysfunction in hemangioma and provide new insights into the cellular interactions underlying HEM regression. Importantly, this study reveals a previously unrecognized mechanism by which fibroblast phenotypic remodeling regulates HemEC function through the PPARγ–Hippo signaling axis, thereby expanding current understanding of the stromal–endothelial interactions involved in HEM progression and regression. Nevertheless, this study is primarily based on an in vitro co-culture system and lacks in vivo validation of the PPARγ–Hippo axis during HEM progression. Future investigations incorporating animal models, clinical samples, and spatial transcriptomic approaches will be necessary to further elucidate the spatiotemporal regulatory mechanisms underlying fibroblast–endothelial communication in HEM and to evaluate the translational potential of targeting this signaling axis.

## Conclusion

In summary, this study reveals a previously unrecognized stromal–endothelial regulatory axis in hemangioma. We demonstrate that PPARγ-driven adipogenic differentiation of fibroblasts suppresses hemangioma endothelial cell proliferation, migration, and angiogenic capacity while promoting apoptosis. Mechanistically, this effect is mediated through activation of the Hippo-YAP/TAZ signaling pathway in endothelial cells. Importantly, pharmacological inhibition of Hippo signaling reverses these effects, further confirming the functional significance of this pathway. Collectively, our findings provide new insights into microenvironment-dependent regulation of HEM progression and suggest that targeting the PPARγ–Hippo signaling axis may represent a promising therapeutic strategy for hemangioma.

## Materials and Methods

The scRNA-seq data were processed and analyzed using the Seurat package, encompassing quality control, dimensionality reduction, clustering, and cell type annotation. Transcriptome sequencing was performed on co-cultured cells following standard library preparation and sequencing protocols. Bioinformatic analyses included differential expression profiling, functional enrichment, and feature gene selection through machine learning approaches. For detailed methodologies, please refer to the Supplementary Methods section.

### Cultivation and grouping of cell lines

Human lung FB cell line HFF-1 (STM-CL-5013, Stemrecell, China) and human HemEC line HemECs (Bio-133629, Biobw, China) were cultured in DMEM high-glucose medium (11965092, Gibco, USA) supplemented with 10% fetal bovine serum (FBS, A5669701, Gibco, USA) and 1% penicillin-streptomycin (15140148, Gibco, USA). Both cell lines were maintained at 37 °C in a humidified incubator with 5% CO₂ (Thermo Scientific, USA), with a subculturing ratio of 1:3 and media changes every two days.

A non-contact co-culture system was established using Transwell inserts with a pore size of 0.4 μm (3412, Corning, USA). Adipogenically induced HFF-1 cells (lower chamber) were co-cultured with HemECs (upper chamber). HFF-1 cells were seeded at a density of 5 × 10⁵ cells per well in the lower chamber, while HemECs were seeded at a density of 1 × 10⁵ cells per well in the upper chamber of a six-well plate. Each cell type was maintained in its respective medium under conditions of 37 °C and 5% CO₂. The experiment was divided into three groups: Control group (uninduced HFF-1 cells), Control + Hippo-on group (uninduced HFF-1 cells with HemECs treated with 10 μM dihydrexidine [DHX; D5814, Sigma-Aldrich, Germany] [53]), Control + Hippo-off group (uninduced HFF-1 cells with HemECs treated with 1 μM XMU-MP-1 [S8334, Selleck Chemicals, USA]), Adipogenesis group (HFF-1 cells post-induction), and Adipogenesis+Hippo-off group (Adipogenesis conditions with the addition of 1 μM XMU-MP-1 to HemECs), with three biological replicates per group. Co-culture duration was set at 48 h.

HFF-1 cells were seeded in six-well plates at a density of 5 × 10⁵ cells per well until reaching approximately 70% confluency. Lipofectamine 3000 transfection reagent (L3000075, Invitrogen, USA) was used to transfect the synthesized small interfering RNA (siRNA provided by RiboBio, China), targeting PPARγ for silencing with si-PPARG and using si-NC as the negative control. The final concentration of siRNA added to each well was 50 nM. Following a transfection period of 48 h, subsequent adipogenic differentiation induction and co-culture experiments were conducted. Three si-PPARG sequences were designed as shown in Table S1; the sequence demonstrating the highest silencing efficiency was selected for further experiments based on RT-qPCR and Western blot analysis.

Adipogenic differentiation of HFF-1 cells was induced using a specific cocktail, and lipid accumulation was assessed through Oil Red O staining, LipidTOX fluorescence, and related quantification. Functional assays including CCK-8, EdU, colony formation, wound healing, Transwell, and tube formation were performed to evaluate viability, proliferation, migration, invasion, and angiogenesis of HemECs. Protein and gene expression analyses involved Western blot, flow cytometry for cell cycle and apoptosis, RT-qPCR, nuclear-cytoplasmic fractionation, and immunofluorescence staining, with antibody details and primer sequences provided in Table S2 and Table S3, respectively. Detailed experimental procedures are described in the Supplementary Methods.

### Statistical Analysis

All data were derived from at least three independent experiments and are presented as the mean ± standard deviation (Mean ± SD). Comparisons between two groups were conducted using independent sample t-tests; for comparisons involving three or more groups, one-way analysis of variance (ANOVA) was employed. If ANOVA indicated significant differences, Tukey’s HSD test was subsequently applied for post-hoc analysis. In cases where data did not meet normal distribution or homogeneity of variance assumptions, the Mann-Whitney U test or Kruskal-Wallis H test was used. All statistical analyses were performed using GraphPad Prism 9.5.0 (GraphPad Software, Inc.) and R 4.2.1 (R Foundation for Statistical Computing). A two-tailed *p*-value < 0.05 was considered statistically significant, while *p* ≥ 0.05 was regarded as not statistically significant.

## Supplementary information

Revised Supplementary Tables and Figures

Supplementary Methods

Original Data

## Data Availability

All data generated or analyzed during this study are included in this article and/or its supplementary material files. Further enquiries can be directed to the corresponding author.
