# Supplementary material for: Fibroblast adipogenesis restricts hemangioma growth through a PPARγ/Hippo-YAP/TAZ axis that promotes endothelial apoptosis and cell cycle arrest
Source: Cell Death Discov. 2026 Jun 17;12:347. doi: 10.1038/s41420-026-03176-x (PMC13507330; doi:10.1038/s41420-026-03176-x)
Supplement: Supplementary file 1 [file 41420_2026_3176_MOESM1_ESM.docx]

**Table S1. siRNA primer sequence.**

| **Gene Name** | **siRNA Sequences(5’- 3’)** |
| --- | --- |
| si-NC (Human) | Guide: 5’-CTTCGTTCAGTATGTTAATCGT-3’ |
|  | Passenger: 5’-CAGAAGAATGGTACAAATCCAAG-3’ |
| si-PPARG-1 (Human) | Guide: 5’-UAAGGAAAGGGAAUUAAGCAG-3’ |
|  | Passenger: 5’-GCUUAAUUCCCUUUCCUUAGA-3’ |
| si-PPARG-2 (Human) | Guide: 5’-UUUAGUGUUGGCUUCUUUCAA-3’ |
|  | Passenger: 5’-GAAAGAAGCCAACACUAAACC-3’ |
| si-PPARG-3 (Human) | Guide: 5’-UAUUUGUGGUUUAGUGUUGGC-3’ |
|  | Passenger: 5’-CAACACUAAACCACAAAUAUA-3’ |

**Table S2. Western blot antibody information.**

| **Target Name** | **Manufacturer** | **Item Number** | **Dilution Ratio** |
| --- | --- | --- | --- |
| Phospho-TAZ (Ser66) (Rabbit) | Abmart | TA4316 | 1:2000 |
| TAZ (Rabbit) | Abmart | PK56462 | 1:2000 |
| TEAD1 (Rabbit) | Abmart | T59103 | 1:2000 |
| Phospho-YAP1 (S127) (Rabbit) | Abmart | T55743 | 1:10000 |
| YAP (Mouse) | Abmart | M027159 | 1:2000 |
| PARIS (Rabbit) | Abcam | ab130867 | 1:2000 |
| PGC-1α (Rabbit) | Abcam | ab191838 | 1:2000 |
| PPAR gamma (Rabbit) | Abmart | T58124 | 1:2000 |
| MST1/2 (Rabbit) | Abmart | TP70673 | 1:2000 |
| MST1/2 (pT183) (Rabbit) | Abmart | PA4262 | 1:1000 |
| LATS1/2 (Rabbit) | Abmart | PU607847 | 1:1000 |
| Phospho-LATS1/2 (Ser909/Ser872) (Rabbit) | Abmart | TA8163 | 1:1000 |
| Histone H3 (Rabbit) | Abmart | TD6932 | 1:1000 |
| Ki67 (Rabbit) | Abcam | ab16667 | 1:500 |
| PCNA (Rabbit) | Abcam | ab92552 | 1:1000 |
| MMP-9 (Rabbit) | Abcam | ab76003 | 1:1000 |
| E-cadherin (Rabbit) | Abcam | ab314063 | 1:1000 |
| Bax (Rabbit) | Abcam | ab32503 | 1:1000 |
| Bcl-2 (Rabbit) | Abcam | ab182858 | 1:2000 |
| Cleaved Caspase-3 (Rabbit) | Abcam | ab214430 | 1:1000 |
| β-Actin (Rabbit) | Abmart | P30002 | 1;2000 |
| GAPDH (Rabbit) | Abmart | P30008 | 1:1000 |

Note: Antibodies purchased from Abcam, UK or Abmart, China.

**Table S3. RT-qPCR** **primer sequence.**

| **Gene Name** | **Primer Sequence** |
| --- | --- |
| PPARG (Human) | Forward: CCAGAAGCCTGCATTTCTGC |
|  | Reverse: CACGGAGCTGATCCCAAAGT |
| GAPDH (Human) | Forward: AATGGGCAGCCGTTAGGAAA |
|  | Reverse: GCGCCCAATACGACCAAATC |

**Figure S1. Quality control, filtering, and clustering of scRNA-seq data.**

Note: (A) Violin plots showing the distribution of gene number per cell (nFeature_RNA), mRNA molecule counts (nCount_RNA), and the percentage of mitochondrial genes (percent.mt); (B) Scatter plots showing the correlation between nCount_RNA and percent.mt, and between nCount_RNA and nFeature_RNA after filtering; (C) Cell cycle states of each cell in the scRNA-seq dataset, with S.Score representing the S phase and G2M.Score representing the G2/M phase; (D) Distribution of standard deviations of PCs, where important PCs exhibit higher standard deviations; (E) Clustering outcomes at different resolutions visualized using the Clustree package.

**Figure S2. Specific gene expression profiles and intercellular communication across cell types.**

Note: (A) t-SNE expression maps of marker genes across eight cell subpopulations, where deeper blue indicates higher average expression; (B) Circos plots showing intercellular communication among ten cell subpopulations, with line thickness representing the strength of signaling interactions.

**Figure S3. Construction and validation of the FB adipogenic differentiation induction system.**

Note: (A) Workflow of FB adipogenic differentiation induction, created in BioRender; (B) Morphological changes of HFF-1 cells after adipogenic induction observed under a microscope (scale bar: 50 μm); (C) ORO staining showing lipid droplet accumulation in HFF-1 cells (scale bar: 50 μm); (D) Quantitative analysis of ORO OD values; (E) LipidTOX staining detecting lipid droplets in HFF-1 cells (scale bar: 25 μm); (F) IF analysis of actin filament (ACTA2) expression changes in HFF-1 cells (scale bar: 25 μm); (G) Western blot analysis of PARIS, PGC-1α, and PPARγ protein expression in HFF-1 cells and corresponding quantitative analysis. All experiments were performed in triplicate. *** indicates *p* < 0.001, **** *p* < 0.0001 compared between groups.

**Figure S4. Quality assessment and differential expression analysis of HFF-1 and HemEC transcriptome data.**

Note: (A) PCA showing sample clustering of HFF-1 transcriptome data; (B) Volcano plot displaying DEGs in HFF-1 cells (filtering criteria: |logFC| > 1 and adjusted *p* value < 0.05); (C) Heatmap showing the top 30 upregulated and downregulated DEGs in HFF-1 cells; (D) PCA showing sample clustering of HemEC transcriptome data; (E) Volcano plot displaying DEGs in HemECs (filtering criteria: |logFC| > 1 and adjusted *p* value < 0.05); (F) Heatmap showing the top 30 upregulated and downregulated DEGs in HemECs. Control group, n = 3; Adipogenesis group, n = 3.

**Figure S5. Alterations in Hippo pathway activity and their effects on YAP/TAZ subcellular localization.**

Note: (A) Western blot analysis of YAP, p-YAP, TAZ, p-TAZ, and TEAD protein expression in HemECs; (B) Western blot analysis of MST1/2, p-MST1/2, LATS1/2, and p-LATS1/2 protein expression in HemECs; (C) Western blot analysis of YAP expression in cytoplasmic and nuclear fractions of HemECs; (D) IF staining showing nuclear localization of YAP and TAZ in HemECs (scale bar: 50 μm). All experiments were performed in triplicate. ** indicates *p* < 0.01, *** *p* < 0.001, **** *p* < 0.0001 compared between groups.
