# Supplementary material for: Fibroblast adipogenesis restricts hemangioma growth through a PPARγ/Hippo-YAP/TAZ axis that promotes endothelial apoptosis and cell cycle arrest
Source: Cell Death Discov. 2026 Jun 17;12:347. doi: 10.1038/s41420-026-03176-x (PMC13507330; doi:10.1038/s41420-026-03176-x)
Supplement: Supplementary file 2 [file 41420_2026_3176_MOESM2_ESM.docx]

**Materials and Methods**

**Download and Analysis of scRNA-seq Data from Tumor Tissue Samples of a Patient with HEM**

The dataset GSE188515 was downloaded from the Gene Expression Omnibus (GEO) database (http://www.ncbi.nlm.nih.gov/geo/), which includes scRNA-seq data from tumor core tissue samples of one patient with HEM.

Data analysis was performed using the 'Seurat' package in R. Quality control criteria were set as 200 < nFeature_RNA < 5000 and percent.mt < 20, leading to the selection of the top 2000 highly variable genes. To reduce the dimensionality of the scRNA-seq dataset, Principal Component Analysis (PCA) was conducted based on these highly variable genes. The ElbowPlot function in 'Seurat' was employed to select the top 20 principal components (PCs) for downstream analysis. Cell subclusters were identified using Seurat's FindClusters function with a default resolution setting (res=1). Subsequently, nonlinear dimensionality reduction was performed on the scRNA-seq data using the t-SNE algorithm. Marker genes for various cell subclusters were identified through 'Seurat', and cells were annotated based on lineage-specific marker genes, in conjunction with online resources such as CellMarker. Additionally, cell communication analysis was conducted using the 'CellChat' package in R.

**High-throughput Transcriptome Sequencing**

The Control group (n=3) and Adipogenesis group (n=3) HFF-1 and HemECs cells were retrieved from the co-culture system. Total RNA was extracted using TRIzol reagent, following the manufacturer's instructions. The RNA purity was assessed with a NanoDrop 2000 spectrophotometer (Thermo Fisher Scientific, USA), with an A260/A280 ratio in the range of 1.8–2.0 considered acceptable. RNA integrity was evaluated using an Agilent 2100 Bioanalyzer (Agilent Technologies, USA), and samples with an RIN value greater than 7.0 proceeded to library preparation.

Qualified samples were submitted to a professional high-throughput sequencing platform for library preparation and sequencing. Libraries were prepared using the Illumina TruSeq Stranded mRNA Library Prep Kit (RS-122-2103, Illumina, USA). The libraries were quantified by qPCR and assessed for fragment length with an Agilent Bioanalyzer 2100. Sequencing was performed on the Illumina NovaSeq 6000 platform in paired-end 150 bp mode (PE150), yielding at least 6 Gb of data per sample.

The raw sequencing data were evaluated for quality using FastQC (v0.11.9), and adapter sequences along with low-quality reads were removed using Trimmomatic (v0.39). High-quality reads were aligned to the human reference genome GRCh38 (GENCODE Release 39) using STAR software (v2.7.10a) under default parameters. Gene-level counts were generated using featureCounts (v2.0.1), producing a raw count matrix for downstream analysis.

**Analysis of Transcriptome Sequencing Data**

Upon importing the expression matrix into R (v4.2.2), sample normalization was conducted using the DESeq2 package (v1.38.3). PCA was performed using the prcomp function, and a two-dimensional scatter plot was visualized with ggplot2 (v3.4.4). Differential expression analysis was executed using the limma package (v3.54.2) in R. Post-normalization data were transformed via voom for linear modeling, with selection criteria set at |logFC| > 1 and adjusted *p* value < 0.05; results were displayed through a volcano plot generated by ggplot2. Differentially expressed genes (DEGs) were subjected to Gene Ontology (GO) and Kyoto Encyclopedia of Genes and Genomes (KEGG) pathway enrichment analysis using the clusterProfiler package (v4.6.2). The enrichGO and enrichKEGG functions were employed, specifying 'Homo sapiens' as the species and setting a q-value threshold below 0.05 for significant enrichment. Results were visualized using the barplot and dotplot functions.

To further identify key DEGs with diagnostic or predictive value, feature gene selection was conducted based on differential expression analysis using LASSO regression and Support Vector Machine Recursive Feature Elimination (SVM-RFE). LASSO regression analysis was performed with the glmnet package (v4.1-8) in R, constructing a logistic regression model with the expression matrix of DEGs as independent variables and sample grouping as dependent variables, determining the optimal penalty parameter lambda via 10-fold cross-validation, and extracting feature genes with non-zero coefficients at this optimal lambda. SVM-RFE analysis was implemented using the e1071 package (v1.7-13) in conjunction with the caret package (v6.0-94), constructing an SVM classification model and applying recursive feature elimination to progressively select genes contributing most significantly to model performance. The union of gene sets derived from both methods was considered as candidate key genes, which were then submitted to the STRING database (https://string-db.org/) with 'Homo sapiens' set as the species and a minimum interaction score threshold of 0.400 to construct a protein-protein interaction (PPI) network, facilitating further exploration of potential biological functions and molecular mechanisms.

**Cultivation and Grouping of Cell Lines**

Human lung FB cell line HFF-1 (STM-CL-5013, Stemrecell, China) and human HemEC line HemECs (Bio-133629, Biobw, China) were cultured in DMEM high-glucose medium (11965092, Gibco, USA) supplemented with 10% fetal bovine serum (FBS, A5669701, Gibco, USA) and 1% penicillin-streptomycin (15140148, Gibco, USA). Both cell lines were maintained at 37°C in a humidified incubator with 5% CO₂ (Thermo Scientific, USA), with a subculturing ratio of 1:3 and media changes every two days.

A non-contact co-culture system was established using Transwell inserts with a pore size of 0.4 μm (3412, Corning, USA), wherein adipogenic-induced HFF-1 cells (lower chamber) were co-cultured with HemECs (upper chamber). HFF-1 cells were seeded at a density of 5 × 10⁵ cells per well in the lower chamber, while HemECs were seeded at a density of 1 × 10⁵ cells per well in the upper chamber of a six-well plate. Each cell type was maintained in its respective medium under conditions of 37°C and 5% CO₂. The experiment was divided into three groups: Control group (uninduced HFF-1 cells), Control + Hippo-on group (uninduced HFF-1 cells with HemECs treated with 10 μM dihydrexidine [DHX; D5814, Sigma-Aldrich, Germany] [56]), Control + Hippo-off group (uninduced HFF-1 cells with HemECs treated with 1 μM XMU-MP-1 [S8334, Selleck Chemicals, USA]), Adipogenesis group (HFF-1 cells post-induction), and Adipogenesis+Hippo-off group (Adipogenesis conditions with the addition of 1 μM XMU-MP-1 to HemECs), with three biological replicates per group. Co-culture duration was set at 48 hours.

HFF-1 cells were seeded in six-well plates at a density of 5 × 10⁵ cells per well until reaching approximately 70% confluency. Lipofectamine 3000 transfection reagent (L3000075, Invitrogen, USA) was used to transfect the synthesized small interfering RNA (siRNA provided by RiboBio, China), targeting PPARγ for silencing with si-PPARG and using si-NC as the negative control. The final concentration of siRNA added to each well was 50 nM. Following a transfection period of 48 hours, subsequent adipogenic differentiation induction and co-culture experiments were conducted. Three si-PPARG sequences were designed as shown in Table S1; the sequence demonstrating the highest silencing efficiency was selected for further experiments based on RT-qPCR and Western blot analysis.

**Induction of Adipogenic Differentiation Experiment**

HFF-1 cells in the logarithmic growth phase were seeded into a 6-well plate at a density of 1 × 10⁵ cells per well. Once the cells adhered, the Adipogenesis group was treated with an adipogenic induction medium containing insulin (5 μg/mL, I2643, Sigma-Aldrich, USA), dexamethasone (1 μM, 265005, Sigma-Aldrich, USA), IBMX (0.5 mM, I5879, Sigma-Aldrich, USA), and indomethacin (60 μM, I8280, Sigma-Aldrich, USA). The induction period lasted for 7 days, with fresh induction medium replaced every 48 hours. The Control group received an equal volume of basal medium without inducers. On day 7 of induction, the morphology of HFF-1 cells in both the Control and Adipogenesis groups was observed under an inverted microscope (Nikon Eclipse Ti-S, Japan), documenting the number, morphology, and distribution characteristics of lipid droplets within the cells.

**Oil Red O (ORO) Staining and Optical Density (OD) Analysis**

After the induction of HFF-1 cells, they were washed three times with PBS (P1020, Solarbio, China) and fixed with 4% paraformaldehyde (P1110, Solarbio, China) for 30 minutes. Subsequently, the cells were treated with 60% isopropanol (I112011, Aladdin, China) for 5 minutes before being stained with freshly prepared ORO solution (G1262, Solarbio, China) for 10 minutes. Following staining, images were captured using a microscope. For quantitative analysis of lipid droplets, the dye was eluted with 100% isopropanol. The eluate was collected and its OD was measured at a wavelength of 500 nm using a microplate reader (BioTek Epoch 2, USA), reflecting the level of lipid accumulation.

**LipidTOX Lipid Droplet Immunofluorescence (IF) Staining**

Fluorescence staining of lipid droplets was performed using LipidTOX™ Red Neutral Lipid Stain (H34477, Thermo Fisher Scientific, USA). HFF-1 cells were first washed three times with PBS, followed by the addition of staining solution containing 1× LipidTOX working solution (dilution ratio 1:1000) and incubated in the dark for 30 minutes. Post-staining, cells were washed twice with PBS and counterstained with DAPI (D1306, Thermo Fisher Scientific, USA) for nuclear visualization. Images were captured using a fluorescence microscope (Leica DMi8, Germany) to observe the distribution of lipid droplets.

**IF Staining of ACTA2 Filaments**

For ACTA2 filament IF staining, HFF-1 cells were fixed and then lysed with 0.2% Triton X-100 (T8200, Solarbio, China) for 10 minutes. Blocking was performed with 5% BSA (SW3015, Solarbio, China) at room temperature for 30 minutes. Subsequently, anti-ACTA2 primary antibody (ab7817, Abcam, UK; dilution 1:200) was added and incubated overnight at 4°C. The next day, an AF488-conjugated goat anti-mouse secondary antibody (A0428, Beyotime, China; dilution 1:500) was applied and incubated in the dark for 1 hour. Nuclear staining was conducted using DAPI, and images were acquired under a fluorescence microscope.

**Cell Counting Kit-8 (CCK-8) Assay**

Following a 24-hour co-culture period, the viability of HemECs was assessed using the CCK-8 (C0037, Beyotime, China). A volume of 10 μL CCK-8 reagent was added and incubated at 37°C for 2 hours. Subsequently, absorbance was measured at a wavelength of 450 nm using a microplate reader (BioTek Epoch 2, USA), reflecting cellular activity.

**EdU Proliferation Assay**

The proliferation capacity of HemECs was assessed using the Click-iT™ EdU Imaging Kit (C0071S, Beyotime, China). HemECs were incubated with 10 μM EdU for 2 hours, followed by fixation, permeabilization, Click reaction, and DAPI counterstaining according to the kit instructions. The number of EdU-positive cells was then observed under a fluorescence microscope (Zeiss Axio Observer, Germany) to calculate the proliferation rate.

**Colony Formation Assay**

HemECs were seeded at a density of 500 cells per well in a 6-well plate and cultured continuously for 14 days, with the medium being replaced every two days. Following colony formation, the cells were fixed using 4% paraformaldehyde (P6148, Sigma-Aldrich, USA) and stained with 0.1% crystal violet solution (V5265, Sigma-Aldrich, USA) for 10 minutes. After thorough washing, images were captured for documentation. Colony numbers were quantified using ImageJ software (NIH, USA).

**Western Blot**

Total protein was extracted from cells using RIPA lysis buffer (P0013B, Beyotime, China) containing 1% PMSF, following the manufacturer's protocol. The supernatant of the samples was collected, and the total protein concentration of each sample was determined using a BCA Protein Assay Kit (P0011, Beyotime, China). Protein concentrations were adjusted to 1 μg/μL, with each sample having a volume of 100 μL. Samples were boiled at 100 °C for 10 minutes to denature the proteins and stored at -80 °C until further use. Based on the target protein band size, an 8%-12% SDS-PAGE gel was prepared. Fifty micrograms of protein from each sample were loaded into each lane using a micropipette. Electrophoresis was conducted at a constant voltage of 80 V, transitioning to 120 V for 2 hours to achieve separation. Proteins were then transferred onto a PVDF membrane (1620177, Bio-Rad, USA) using a constant current of 250 mA for 90 minutes.

The membrane was blocked with 5% non-fat milk in 1×TBST at room temperature for 1 hour. After discarding the blocking solution, the membrane was washed with 1×TBST for 10 minutes. Primary antibodies (details in Table S2) were incubated overnight at 4 °C. The membrane was washed three times with 1×TBST, each wash lasting 5 minutes. HRP-conjugated goat anti-rabbit IgG (ab6721, dilution: 1:5000, Abcam, UK) or goat anti-mouse IgG (ab6789, dilution: 1:5000, Abcam, UK) secondary antibodies were applied and incubated at room temperature for 1 hour. The membrane was washed three times with TBST buffer at room temperature for 5 minutes each time. It was then immersed in ECL reagent (1705062, Bio-Rad, USA) and incubated at room temperature for 1 minute. Excess liquid was removed before covering the membrane with plastic wrap for band exposure imaging using an Image Quant LAS 4000C system (GE Healthcare, USA). GAPDH served as an internal control for total cellular protein levels; relative protein expression levels were determined by comparing the grayscale values of target bands to those of the internal reference bands. Each experiment was performed in triplicate.

**Detection of Cell Cycle and Apoptosis via Flow Cytometry**

Following co-culture, HemECs were digested and collected using 0.25% trypsin (T6567, Sigma-Aldrich, USA). For cell cycle analysis, the cells were washed with PBS (HyClone, USA) and subsequently fixed overnight at 4°C in 70% pre-chilled ethanol (187380, Sigma-Aldrich, USA). Post-fixation, the cells were resuspended in a staining solution containing 50 μg/mL RNase A (R1253, Thermo Fisher Scientific, USA) and 25 μg/mL propidium iodide (PI, P1304MP, Thermo Fisher Scientific, USA), incubated at 37°C in the dark for 30 minutes. Data acquisition was performed using a flow cytometer (BD FACSCanto II, USA), and analysis of the proportions of cells in G0/G1, S, and G2/M phases was conducted using FlowJo software (v10.8.1, BD Biosciences, USA).

For apoptosis detection, an Annexin V-FITC/PI double staining kit (BD Biosciences, USA) was employed. After washing with PBS, cells were resuspended in 100 μL of 1× binding buffer to which 5 μL of Annexin V-FITC and 5 μL of PI were added. The mixture was incubated at room temperature in the dark for 15 minutes. Subsequently, 400 μL of binding buffer was added immediately before analysis using the BD FACSCanto II flow cytometer. The data were analyzed with FlowJo software to determine the proportions of early and late apoptotic cells.

**Wound Healing Assay**

HemECs were seeded in 6-well plates and allowed to reach 90% confluence. A sterile 200 μL pipette tip was used to create a scratch, and PBS was used to wash away cell debris. The cells were then cultured in serum-free medium. Photographs were taken at 0 and 24 hours, and the change in scratch width was measured using ImageJ to calculate the migration rate.

**Transwell Invasion Assay**

The Transwell chambers (8 μm pore size, Corning, USA) were pre-coated with Matrigel matrix (356234, Solarbio, China) and placed in a 24-well plate. HemECs were seeded at a density of 1 × 10⁵ cells per well into the upper chamber, while medium containing 10% FBS (S9020, Solarbio, China) was added to the lower chamber. After a 24-hour incubation period, non-migrated cells were removed using a cotton swab. The cells were then fixed with 4% paraformaldehyde and stained with crystal violet (V5265, Sigma-Aldrich, USA). Invasive cells were observed and counted under a microscope (Nikon Eclipse Ti-S, Japan).

**Tube Formation Assay**

A 96-well plate was pre-coated with Matrigel (C0372, Beyotime, China), with 50 μL added to each well, and incubated at 37°C for 30 minutes. HemECs were seeded onto the Matrigel at a density of 2 × 10⁴ cells per well. After a 6-hour incubation, the formation of tubular structures was observed under an inverted microscope (Olympus CKX53, Japan) and analyzed using the Angiogenesis Analyzer plugin in ImageJ to quantify the number and total length of the tubes formed.

**Detection of Gene Expression Using RT-qPCR**

Total RNA was extracted from cells using the Trizol kit (A33254, Thermo Fisher, USA). Reverse transcription was performed to synthesize cDNA using the reverse transcription kit (RR047A, Takara, Japan). The reaction system was prepared using the SYBR® Premix Ex TaqTM II kit (DRR081, Takara, Japan), and RT-qPCR was conducted in a real-time quantitative PCR instrument (ABI7500, Thermo Fisher, USA). The PCR protocol was designed as follows: initial denaturation at 95°C for 30 seconds, followed by 40 cycles of denaturation at 95°C for 5 seconds and annealing at 60°C for 30 seconds. Subsequently, an extension at 95°C for 15 seconds and at 60°C for 60 seconds was performed, followed by a final extension at 90°C for 15 seconds to generate the amplification curve. GAPDH served as the reference gene. All RT-qPCR reactions were set up in triplicate wells and repeated three times. The relative expression of the target gene between experimental and control groups was calculated using the 2^-ΔΔCt^ method, where ΔΔCT = ΔCt _experimental group_ - ΔCt _control group_, and ΔCt = Ct _target gene_ - Ct _reference gene_. Ct represents the number of amplification cycles required for the fluorescence intensity to reach a predefined threshold during the exponential growth phase. Primer designs are detailed in Table S3.

**Nuclear-Cytoplasmic Fractionation and YAP Localization Assessment**

Nuclear and cytoplasmic proteins from HemECs were extracted using a nuclear and cytoplasmic protein extraction kit (P0027, Beyotime, China), following the manufacturer's instructions. Protein concentrations were determined via the BCA method, and subsequent Western blot analysis was performed. YAP antibody (M027159, 1:2000, Abmart, China), β-Actin (P30002, cytoplasmic control 1:2000, Abmart, China), and Histone H3 (T56587, nuclear control 1:2000, Abmart, China) were used as controls to verify the distribution of YAP between the nucleus and cytoplasm.

**IF Colocalization Experiment**

HemECs were seeded onto coverslips in a 6-well plate and co-cultured for 24 hours. Subsequently, the cells were fixed in 4% paraformaldehyde for 10 minutes and washed three times with PBS. Permeabilization was performed using 0.2% Triton X-100 for 10 minutes. The blocking solution consisted of 5% BSA, and incubation was carried out at room temperature for 30 minutes before adding primary antibodies: YAP (ab52771, Abcam, 1:200) and TAZ (ab242313, Abcam, 1:200), which were incubated overnight at 4°C. The following day, corresponding Alexa Fluor 488 or 555-conjugated secondary antibodies (A0423, A0460, Beyotime, China, 1:500) were added for a one-hour incubation. Nuclei were counterstained with DAPI. Images were captured using a confocal laser scanning microscope (Leica TCS SP8, Germany) and analyzed for localization using ImageJ software.

**Statistical Analysis**

All data were derived from at least three independent experiments and are presented as the mean ± standard deviation (Mean ± SD). Comparisons between two groups were conducted using independent sample t-tests; for comparisons involving three or more groups, one-way analysis of variance (ANOVA) was employed. If ANOVA indicated significant differences, Tukey's HSD test was subsequently applied for post-hoc analysis. In cases where data did not meet normal distribution or homogeneity of variance assumptions, the Mann-Whitney U test or Kruskal-Wallis H test was used. All statistical analyses were performed using GraphPad Prism 9.5.0 (GraphPad Software, Inc.) and R 4.2.1 (R Foundation for Statistical Computing). A two-tailed *p*-value < 0.05 was considered statistically significant, while *p* ≥ 0.05 was regarded as not statistically significant.
