# Supplementary material for: Fibroblast adipogenesis restricts hemangioma growth through a PPARγ/Hippo-YAP/TAZ axis that promotes endothelial apoptosis and cell cycle arrest
Source: Cell Death Discov. 2026 Jun 17;12:347. doi: 10.1038/s41420-026-03176-x (PMC13507330; doi:10.1038/s41420-026-03176-x)
Supplement: Supplementary file 3 [file 41420_2026_3176_MOESM3_ESM.docx]

# uncropped Gels and Blots image of Figure 10E-1.jpg

# uncropped Gels and Blots image of Figure 10E-2.jpg

# uncropped Gels and Blots image of Figure 10E-3.jpg

# uncropped Gels and Blots image of Figure 11C-1.jpg

# uncropped Gels and Blots image of Figure 11C-2.jpg

# uncropped Gels and Blots image of Figure 11C-3.jpg

# uncropped Gels and Blots image of Figure 11F-1.jpg

# uncropped Gels and Blots image of Figure 11F-2.jpg

# uncropped Gels and Blots image of Figure 11F-3.jpg

# uncropped Gels and Blots image of Figure 11F-4.jpg

# uncropped Gels and Blots image of Figure 2E-1.jpg

# uncropped Gels and Blots image of Figure 2E-2.jpg

# uncropped Gels and Blots image of Figure 2E-3.jpg

# uncropped Gels and Blots image of Figure 3D-1.jpg

# uncropped Gels and Blots image of Figure 3D-2.jpg

# uncropped Gels and Blots image of Figure 3D-3.jpg

# uncropped Gels and Blots image of Figure 3G-1.jpg

# uncropped Gels and Blots image of Figure 3G-2.jpg

# uncropped Gels and Blots image of Figure 3G-3.jpg

# uncropped Gels and Blots image of Figure 3G-4.jpg

# uncropped Gels and Blots image of Figure 6C-1.jpg

# uncropped Gels and Blots image of Figure 6C-2.jpg

# uncropped Gels and Blots image of Figure 6F-1.jpg

# uncropped Gels and Blots image of Figure 6F-2.jpg

# uncropped Gels and Blots image of Figure 6F-3.jpg

# uncropped Gels and Blots image of Figure 6F-4.jpg

# uncropped Gels and Blots image of Figure 6F-5.jpg

# uncropped Gels and Blots image of Figure 6G-1 (1).jpg

# uncropped Gels and Blots image of Figure 6G-1 (2).jpg

# uncropped Gels and Blots image of Figure 6G-2 (1).jpg

# uncropped Gels and Blots image of Figure 6G-2 (2).jpg

# uncropped Gels and Blots image of Figure 6G-3(1).jpg

# uncropped Gels and Blots image of Figure 6G-3(2).jpg

# uncropped Gels and Blots image of Figure 7C-1.jpg

# uncropped Gels and Blots image of Figure 7C-2.jpg

# uncropped Gels and Blots image of Figure 8E-1.jpg

# uncropped Gels and Blots image of Figure 8E-2.jpg

# uncropped Gels and Blots image of Figure 8E-3.jpg

# uncropped Gels and Blots image of Figure 9D-1.jpg

# uncropped Gels and Blots image of Figure 9D-2.jpg

# uncropped Gels and Blots image of Figure 9D-3.jpg

# uncropped Gels and Blots image of Figure 9G-1.jpg

# uncropped Gels and Blots image of Figure 9G-2.jpg

# uncropped Gels and Blots image of Figure 9G-3.jpg

# uncropped Gels and Blots image of Figure 9G-4.jpg

# uncropped Gels and Blots image of Figure S3G-1.jpg

# uncropped Gels and Blots image of Figure S3G-2.jpg

# uncropped Gels and Blots image of Figure S3G-3.jpg

# uncropped Gels and Blots image of Figure S3G-4.jpg

# uncropped Gels and Blots image of Figure S5A-1.jpg

# uncropped Gels and Blots image of Figure S5A-2.jpg

# uncropped Gels and Blots image of Figure S5A-3.jpg

# uncropped Gels and Blots image of Figure S5A-4.jpg

# uncropped Gels and Blots image of Figure S5A-5.jpg

# uncropped Gels and Blots image of Figure S5A-6.jpg

# uncropped Gels and Blots image of Figure S5B-1.jpg

# uncropped Gels and Blots image of Figure S5B-2.jpg

# uncropped Gels and Blots image of Figure S5B-3.jpg

# uncropped Gels and Blots image of Figure S5B-4.jpg

# uncropped Gels and Blots image of Figure S5B-5.jpg

# uncropped Gels and Blots image of Figure S5C-1(1).jpg

# uncropped Gels and Blots image of Figure S5C-1(2).jpg

# uncropped Gels and Blots image of Figure S5C-2(1).jpg

# uncropped Gels and Blots image of Figure S5C-2(2).jpg

# uncropped Gels and Blots image of Figure S5C-3(1).jpg

# uncropped Gels and Blots image of Figure S5C-3(2).jpg
